# Mosaic chromosomal alterations in hematopoietic cells and clinical outcomes in patients with multiple myeloma

**DOI:** 10.1038/s41375-024-02396-3

**Published:** 2024-09-02

**Authors:** Simon Husby, Morten Tulstrup, Mads Harsløf, Christian Nielsen, Eva Haastrup, Lene Hyldahl Ebbesen, Mette Klarskov Andersen, Maroulio Pertesi, Christian Brieghel, Carsten U. Niemann, Björn Nilsson, Agoston Gyula Szabo, Niels Frost Andersen, Niels Abildgaard, Annette Vangsted, Kirsten Grønbæk

**Affiliations:** 1 Department of Hematology, Rigshospitalet, Denmark, Copenhagen N, Denmark; 2 Biotech Research and Innovation Centre, BRIC, University of Copenhagen, Copenhagen, Denmark; 3 Department of Clinical Immunology, Odense University Hospital, Odense, Denmark; 4 Centre for Cellular Immunotherapy of Haematological Cancer, Odense, Denmark; 5 Department of Clinical Immunology, Rigshospitalet, Copenhagen, Denmark; 6 Department of Hematology, Aarhus University Hospital, Aarhus, Denmark; 7 Department of Clinical Genetics, Rigshospitalet, Copenhagen, Denmark; 8 Division of Hematology and Transfusion Medicine, Lund University, Lund, Sweden; 9 Hematology Research Unit, Department of Hematology, Odense University Hospital, and Department of Clinical Research, University of Southern Denmark, Odense, Denmark; 10 Department of Clinical Medicine, Faculty of Health and Medical Sciences, University of Copenhagen, Copenhagen, Denmark

**Keywords:** Translational research, Genetics research

## Abstract

Mosaic chromosomal alterations (mCAs) in hematopoietic cells increase mortality and risk of hematological cancers and infections. We investigated the landscape of mCAs and their clinical consequences in 976 patients with multiple myeloma undergoing high-dose chemotherapy and autologous stem cell support (ASCT) with median 6.4 years of follow-up. mCAs were detected in the stem cell harvest product of 158 patients (16.2%). Autosomal aberrations were found in 60 patients (6.1%) and affected all chromosomes. Loss of chromosome X was found in 51 females (12.7%) and loss of chromosome Y in 55 males (9.6%). Overall survival and progression were similar between carriers of autosomal mCAs and non-carriers. In contrast, female patients with loss of the X chromosome had longer overall survival (age-adjusted[a.a.] HR 0.54, 95% CI 0.32–0.93, *p* = 0.02), lower risk of progression (a.a. HR 0.55, 95% CI 0.35–0.87; *p* = 0.01), and better post-transplant response (higher degree of complete response (CR) or very good partial response (VGPR)). The reason for this substantial effect is unknown. Additionally, myeloma clones in the stem cell product was confirmed by mCA analysis in the few patients with multiple mCAs (*n* = 12 patients). Multiple mCAs conferred inferior overall survival (a.a. HR 2.0, 95% CI 1.02–3.84; *p* = 0.04) and higher risk of myeloma progression (a.a. HR 3.36, 95% CI 1.67–6.81; *p* < 0.001), which is presumed to be driven by suspected myeloma contaminants.

## Highlights

- Mosaic chromosomal alterations (mCAs) are found in the hematopoietic cells in 16% of patients with multiple myeloma undergoing ASCT.
- Female patients with loss of chromosome X have a significant superior response to ASCT and improved overall survival despite being older.

## Introduction

Hematopoietic stem cells (HSC) in healthy human individuals acquire genetic alterations during a normal life span [1]. These age-related clonal events include single-nucleotide variants (SNVs), small insertions/deletions (indels) of few basepairs, gains and losses of larger chromosomal material, as well as copy-number neutral loss of heterozygosity (CN-LOH). Large alterations (>50 kb) are referred to as mosaic chromosomal alterations (mCAs), while SNVs and indels are termed clonal hematopoiesis of indeterminate potential (CHIP). Novel bioinformatic methods have made it possible to identify low-frequency cell populations with mCAs in DNA samples using genome-wide SNP arrays [2]. The occurrence of mCAs is highly age-related, associated with hematological malignancies, and increased risks of infections [3].

Several groups have examined the occurrence of CHIP in patients with multiple myeloma (MM) [4–7]. CHIP variants are common in patients with MM with a prevalence of 21–23% in patients receiving autologous stem cell transplant (ASCT). The most recent, and largest study by Mouheddine et al. found that CHIP was associated with increased age, risk of recurrent bacterial infections, but not with decreased overall or progression-free survival. Prior studies have primarily made use of targeted next-generation sequencing, which cannot detect smaller chromosomal aberrations.

The presence of mCAs in the blood cells of patients with MM has, to our knowledge, not been examined. We therefore investigated the prevalence and characteristics of mCAs in patients with MM undergoing ASCT. Furthermore, we report the long-term clinical impact of mCAs in a national myeloma cohort with comprehensive follow-up data.

## Methods

### Patients and samples

Post-induction peripheral blood stem cell apheresis material from 980 patients with multiple myeloma who were intended for ASCT and part of the Danish National Myeloma ASCT cohort. Samples were collected from the respective departments of clinical immunology (Copenhagen, Aarhus, and Odense) and purified for DNA. Samples were obtained from stem cell harvest performed between 1995 and 2016. In the 19 patients with multiple autologous transplants, the earliest available sample was used. Samples were analyzed at deCODE genetics, Reykjavik, Iceland with a custom Illumina deCODE SNP array as previously described [8, 9]. Genetic analyses were approved by the Ethical Committee in the Capital Region of Denmark (approval no. H-16032570) and access to registry data was approved by the National Danish Committee on Research Ethics (journal no. 72153). In accordance with national Danish law and approval form the National Danish Committee, informed consent was waived for use of blood samples and registry data for research, unless patients were enlisted in the National Tissue Research Registry (i.e., possibility to ‘opt-out’ of research participation). No patients in this study were enrolled in this ‘opt-out’ registry, and thus all could be legally included in the analyses. All methods in this manuscript were performed in accordance with national regulations and relevant guidelines.

### Detection of mosaic chromosomal alterations

The raw IDAT files were converted to gtc files using Illumina’s GenCall algorithm and then combined into a single vcf file using gtc2vcf with the—adjust-clusters option and otherwise default parameters (https://github.com/freeseek/gtc2vcf). Genotype phasing was done using shapeit4 [10] with the 1000 Genomes project low coverage reference panel of 2504 samples, and mCA calling performed using the Mosaic Chromosomal Alterations (MoChA) software (https://github.com/freeseek/mocha) with disabled BAF and LRR adjustment and otherwise default parameters as recommended on the MoChA GitHub page [2, 11]. At all steps, we used GRCh37 manifest and reference files. Sample and callset filtering, including detection of mosaic loss of the X and Y chromosomes was done using the recommended filtering pipeline and default parameters as recommended by the MoChA authors. MoChA enables identification of small interstitial aberrations near the centromere of chromosome 14 (positions 18,000,000–23,000,000). These events most likely represent T-cell receptor rearrangements and germline segmental duplications [12], thus identifying mature T-cells in the sample and not clonally expanded myeloid cells. We identified 16 such small aberrations near the centromere of chromosome 14, which we excluded from further analyses, as done in some prior mCA studies [2].

### Clinical characteristics and outcomes

Clinical annotation was available on 825 patients of the 976 patients with available genomic material. Prospectively collected clinical data was retrieved from the Danish Multiple Myeloma Registry [13, 14] (DaMyDa; includes baseline MM characteristics, biochemical data, dates of diagnosis, ASCT, progression, and death) and the Danish National Registry of Patients (DNPR; includes information on all hospital admission and outpatient visits, and associated diseases/treatments by ICD-10 code) [15]. Details on use of data from the DaMyDa and a list of analyzed ICD-10 codes from the DNPR can be found in Supplemental Note 1. All biochemical data were from time of MM diagnosis. A total of 72 patients had no prospectively collected registry data, but clinical data available from a retrospective study review of medical records of patients diagnosed from 1995 to 2004 [16].

### Statistical analyses

Wilcoxon rank-sum and Student’s *t*-test were used for comparison of baseline clinical data in patients with and without detected mCA’s. The association between mCAs and bone marrow infiltration was investigated using logistic regression with mCA as the outcome and bone marrow infiltration divided into quartiles as explanatory variable with adjustment for age at the date of harvest and sex. Myeloma/M-spike protein (M protein) was investigated similarly, but with adjustment for type of M protein (IgG, IgA, Other), and patients with light-chain M protein were excluded from analyses. Associations with hemoglobin level were investigated with hemoglobin as a continuous outcome and mCA as explanatory variables, with adjustment for sex and bone marrow infiltration in quartiles. All analyses regarding loss of X or loss of Y, were solely performed with only female or male subjects, respectively.

Time-to-event variables were defined as time from date of stem cell harvest to date of the event of interest. Patients without registered events were censored at last follow-up (January 15, 2022).

Probability of overall survival was estimated with the Kaplan–Meier method. Cumulative incidence of first progression or infection (analyzed separately) was estimated by the Aalen–Johansen estimator considering death as a competing event, and the estimates were compared with Gray’s test. Multivariable Cox proportional hazards regression of overall survival (OS) was carried out first in a baseline model with adjustment for age, age squared (to allow for potential non-linear effects of age), and stratification by sex (due to a modest violation of the proportional hazards assumption by sex). Furthermore, separate models were tested with additional inclusion of essential covariates; year of stem cell harvest (categorized as: before 2005, 2005–2008, 2009–2011, 2012–2017, on the basis of previously published national treatment data [17]), type of induction therapy (categorized as; velcade-cyclophosphamide-dexamethasone, velcade-dexamethasone, velcade-imid-dexamethasone (thalidomide or lenalidomide), vincristine-doxorubicin-dexamethasone, and other treatments), International Staging System (ISS) group, and bone marrow infiltration (categorized into quartiles). Similarly, we analyzed cause-specific hazards of progression and infection while censoring deaths using the same covariates as in OS analyses. Where the proportional hazards assumption was not met for, we allowed for time-dependent effects of the given covariate.

Induction regimen prior to ASCT was also split for use in multivariate analysis in the five major treatment categories.

Associations between number of erythrocyte transfusions in the first year after transplant and mCAs were tested using Wilcoxon rank-sum test. For each statistical test, type of adjustment is specifically mentioned. If not, *p* values are unadjusted. All statistical analyses were performed using R version 3.6.1.

## Results

### Landscape of mCAs in patients with multiple myeloma

In total, mCAs were detected in 158 patients (16.2%) from the National Danish MM cohort, who were all intended for autologous stem cell transplantation. Of these, 106 patients (10.9%) had clonal loss of a sex chromosome with respectively 51 females (12.7% of all females) having loss of chromosome X and 55 males (9.6% of all males) with loss of chromosome Y (Supplementary Table 1). A total of 60 patients (6.1%) had an autosomal mCA. Concurrent autosomal mCA and loss of a sex chromosome were identified in eight patients (0.8%).

**Fig. 1:**
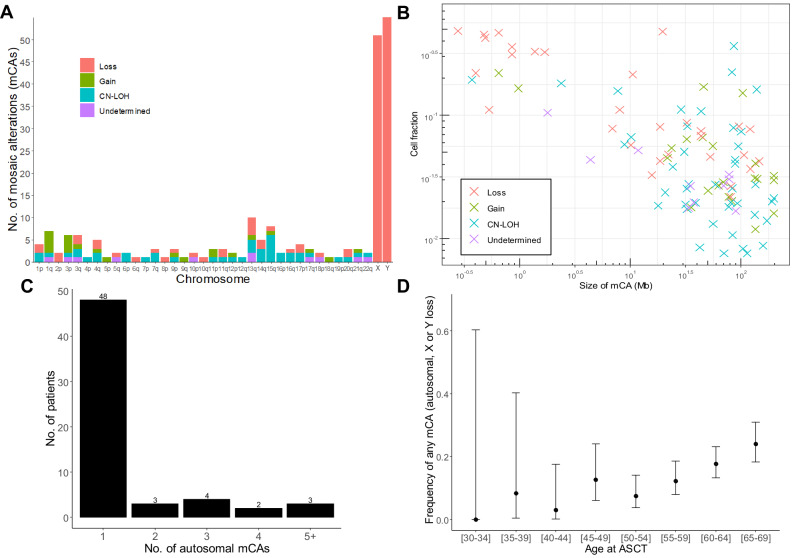
Landscape of mCAs in patients with multiple myeloma. **A** Frequency and type of mCAs per chromosome detected in the cohort. CN-LOH, copy number neutral loss of heterozygosity. **B** Distribution of mCA size and clonal cell fraction. **C** Number of subjects with 1, 2, 3, 4 or 5+ autosomal mCAs. **D** Age distribution of mCA prevalence. Dots show frequency, while bars show 95% confidence interval for the specific subgroup.

The spectrum of autosomal mCAs was diverse with variants identified in all chromosomes (Fig. 1A, Supplementary File ‘Genomic locations of mCAs’). Type of autosomal mCA was classified as 36% CN-LOH, 31% losses, 20% gains, and 13% as undetermined. The genomic size of autosomal mCAs ranged from 206 kb to 198 Mb with the median size being 34.6 Mb (distribution shown in Supplementary Table 2). The estimated fraction of cells with chromosomal alterations was larger for autosomal variants (median cell fraction 4.9%) and loss of chromosome Y (median cell fraction 5.1%) in comparison with loss of chromosome X (median cell fraction 2.5%, Supplementary Fig. 1). This is a technical consequence of the large genomic size of the entire X chromosome (154 Mb, the whole chromosome used for detection by MoChA), compared with the median autosomal variant size of 34.6 Mb and Y chromosome size of 62 Mb, since larger chromosomal alterations are more easily detected at low cell frequencies by MoChA. The far majority of patients (*n* = 48) only had one detectable autosomal mCA, while 12 patients had two or more mCAs (Fig. 1C).

### Clinical characteristics associated with mCAs

**Fig. 2:**
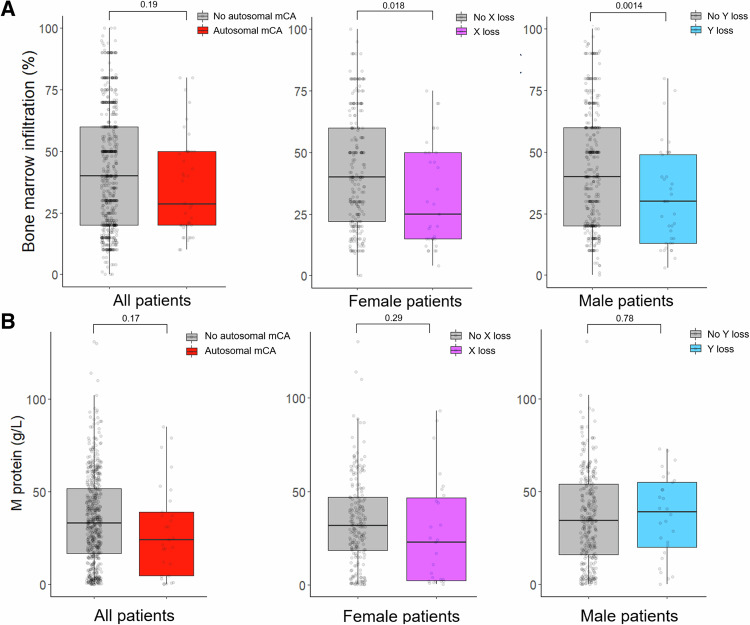
Phenotypic associations with the presence of mCAs. **A** Myeloma bone marrow infiltration at diagnosis and presence of mCA in the stem cell product. **B** M protein level in the peripheral blood at diagnosis and presence of mCA in the stem cell product.

MCA prevalence (both autosomal and loss of a sex chromosome) was highly age-related, with 24% of patients between 65 and 69 years carrying one or more mCAs compared to 3% in 40–44-year-olds (Fig. 1D). Females and males had a similar frequency of autosomal mCAs (Table 1 and Supplementary Table 3; *n* = 25/376 (6.6%) and 35/539 (6.5%), respectively, unadjusted *p* = 1.0). The median myeloma cell infiltration in male patients with loss of chromosome Y was 30% compared to 40% in males without loss of Y (unadjusted *p* = 0.0014, Fig. 2A), and the median infiltration in females with vs. without loss of X was 25% and 40%, respectively (unadjusted *p* = 0.018). To adjust for confounding we fitted logistic regression models with X and/or Y loss as the outcome and bone marrow infiltration divided into quartile groups as explanatory variables together with sex and age at harvest. In separate models for males and females, all quartile groups 2–4 all had lower occurrence of sex chromosome loss than the lower quartile (reference group, Supplementary Table 4), but the overall effect of bone marrow infiltration was not statistically significant (log-likelihood ratio test: *p* = 0.09 for females and *p* = 0.20 for males). When analyzing males and females together (with adjustment for sex) the ORs for sex chromosome loss were 0.95, 0.94 and 0.88 in quartile groups 2, 3, and 4, respectively (log-likelihood ratio test: *p* = 0.022). We observed no significant differences in M protein level at diagnosis between patients with vs. without autosomal mCA, loss of X or loss of Y in univariate (Fig. 2B) or multivariate analyses (Supplementary Table 5). Time from start of induction therapy to ASCT (defined as day of stem cell infusion) did not differ significantly between patients with or without mCAs (Table 1).

**Table 1:** Characteristics of the Danish National Myeloma ASCT cohort.

		No mCA	Autosomal mCA	Loss of X	Loss of Y
No. of patients		817	60	51	55
Age		58.4 (7.6)	61.1 (6.0)	61.5 (7.6)	62.6 (5.3)
Sex (%)	Female	331 (40.5)	25 (41.7)	51 (100.0)	0 (0.0)
	Male	486 (59.5)	35 (58.3)	0 (0.0)	55 (100.0)
Type of M protein	IgA	130 (22.0)	9 (23.7)	8 (23.5)	6 (16.7)
	IgG	365 (61.7)	23 (60.5)	17 (50.0)	23 (63.9)
	Light-chain	55 (9.3)	3 (7.9)	8 (23.5)	4 (11.1)
	Other	42 (7.1)	3 (7.9)	1 (2.9)	3 (8.3)
Hemoglobin [mmol/L]		6.7 (1.2)	6.6 (1.2)	6.8 (1.2)	7.3 (1.1)
β2M [mg/L]		5.0 (5.0)	6.4 (8.7)	4.7 (4.4)	3.8 (3.2)
Albumin [g/L]		36.0 (7.0)	37.1 (6.0)	37.2 (7.2)	35.9 (5.5)
LDH [U/L]		191.5 (98.4)	186.6 (103.9)	190.5 (128.5)	184.2 (71.1)
Stage (ISS)	1	175 (35.3)	14 (37.8)	12 (38.7)	16 (44.4)
	2	187 (37.7)	13 (35.1)	12 (38.7)	13 (36.1)
	3	134 (27.0)	10 (27.0)	7 (22.6)	7 (19.4)
Bone marrow infiltration (%)		43.2 (24.0)	35.9 (20.4)	32.5 (21.5)	31.2 (20.2)
M protein [g/L]		35.4 (24.1)	28.5 (24.9)	27.8 (28.5)	37.1 (22.3)
Days from induction to ASCT		146.8 (180.3)	145.8 (121.9)	160.8 (288.8)	161.3 (159.5)
Induction regimen	CyDex	236 (33.5)	17 (35.4)	9 (22.0)	19 (41.3)
	VimidD	45 (6.4)	3 (6.2)	1 (2.4)	2 (4.3)
	VAD	62 (8.8)	3 (6.2)	3 (7.3)	2 (4.3)
	VCd	173 (24.5)	9 (18.8)	14 (34.1)	7 (15.2)
	Vd	137 (19.4)	11 (22.9)	7 (17.1)	11 (23.9)
	Other	52 (7.4)	5 (10.4)	7 (17.1)	5 (10.9)

Data are presented as means with standard deviation in parenthesis, unless otherwise noted. Of the 976 patients analyzed for mosaic chromosomal alterations, there was clinical annotation on 825 patients. Patients with missing clinical information are only represented in the first line of the table (describing number of patients with autosomal mCA, loss of chromosome X or Y). Eight patients had both an autosomal mCA and loss of a sex chromosome and are thus included in two columns. Therefore, the total fraction of patients from the full cohort represented in this table surpasses 100%. Days from induction to ASCT is counted from the day 1 of first induction therapy to the day of autologous stem cell infusion. Peripheral blood biochemistry data (hemoglobin, beta-2-microglobulin, albumin, lactate dehydrogenase, M protein) and bone marrow infiltration is from time of diagnosis.

*IgA* immunoglobulin A, *IgG* immunoglobulin G, *Other* immunoglobulin E, D or not-determined, *β2M* beta-2-microglobulin, *LDH* lactate dehydrogenase, *ISS* International Staging System, *ASCT* autologous stem cell transplant, *CyDex* cyclophosphamide-dexamethasone, *VimidD* velcade-thalidomide/lenalidomide- dexamethasone, *VAD* vincristine-adriamycine- dexamethasone, *VCd* velcade-cyclophosphamide-dexamethasone, *VD* velcade-dexamethasone, *Other* other induction regimen.

Other baseline parameters; hemoglobin, albumin, lactate dehydrogenase, and International Staging System (ISS) score, were not significantly associated with presence of mCAs (Table 1, Supplementary Table 3). Furthermore, there was no significant association between type of induction regimen and occurrence of mCAs (Supplementary Table 6A–C). High-risk MM cytogenetics by fluorescent in-situ hybridization (FISH) according to Sonneveld P. et al. [18] was not associated with occurrence of autosomal mCAs, loss of X or loss of Y (Supplementary Table 7).

### Associations between mCAs and post-transplant events

**Fig. 3:**
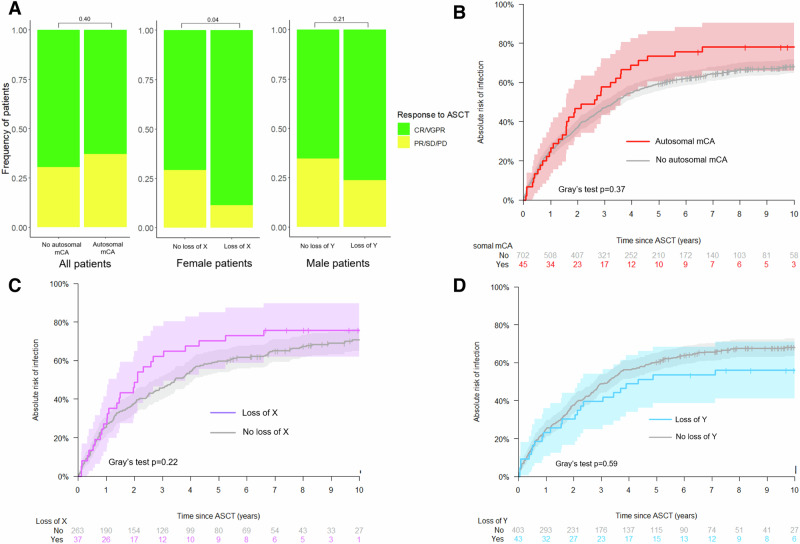
Adverse events after ASCT in patients with mCAs. **A** Treatment response after ASCT. CR/VGPR, complete response/very good partial response; PR/SD/PD, partial response, stable disease, progressive disease. **B** Cumulative incidence of admission with infection for patients with autosomal mCA. **C** Cumulative incidence of admission with infection among female patients with or without loss of chromosome X. **D** Cumulative incidence of admission with infection among male patients with or without loss of chromosome Y.

To investigate if mCAs were associated with adverse events after ASCT, we examined the post-transplant response evaluation, hospital admissions for infection, blood transfusions, and days admitted to hospital in patients from the national myeloma cohort. Best response after ASCT therapy was similar between patients with and without autosomal mCAs (Fig. 3A). Among female patients with clonal loss of chromosome X, 31 (89%) had a favorable response (complete response (CR) or very good partial response (VGPR)) compared to only 181 (70%) of female patients without loss of X (Chi squared test: *p* = 0.04). There was no difference in response between male patients with and without loss of Y chromosome (Chi squared test: *p* = 0.21).

With regards to post-transplant hospital admission for infection, a non-significantly increased cumulative incidence of first infection in patients with autosomal mCAs was observed (Fig. 3B; Gray’s test unadjusted *p* = 0.13). Loss of the X or Y chromosome was not associated with altered risk of hospital admission with infection (Fig. 3C, D; Gray’s test *p* = 0.22 and *p* = 0.59, respectively).

The median number of red blood cell transfusions in the first five years after ASCT was 1 in patients both with and without autosomal mCAs (unadjusted *p* = 0.18). Female patients with loss of chromosome X had a median of 1 (IQR 0–2) blood transfusions compared with 1 (IQR 0–4) for those without loss of chromosome X (unadjusted *p* = 0.09). There was no difference in number of transfusions between male patients with and without loss of the Y chromosome (unadjusted *p* = 0.34).

The number of days admitted to hospital in the first year after ASCT, and year 2–5 after ASCT, was similar in patients with and without autosomal mCAs, loss of chromosome X or loss of chromosome Y (Supplementary Figs. 2–3).

### Loss of sex chromosome X is associated with improved survival

**Fig. 4:**
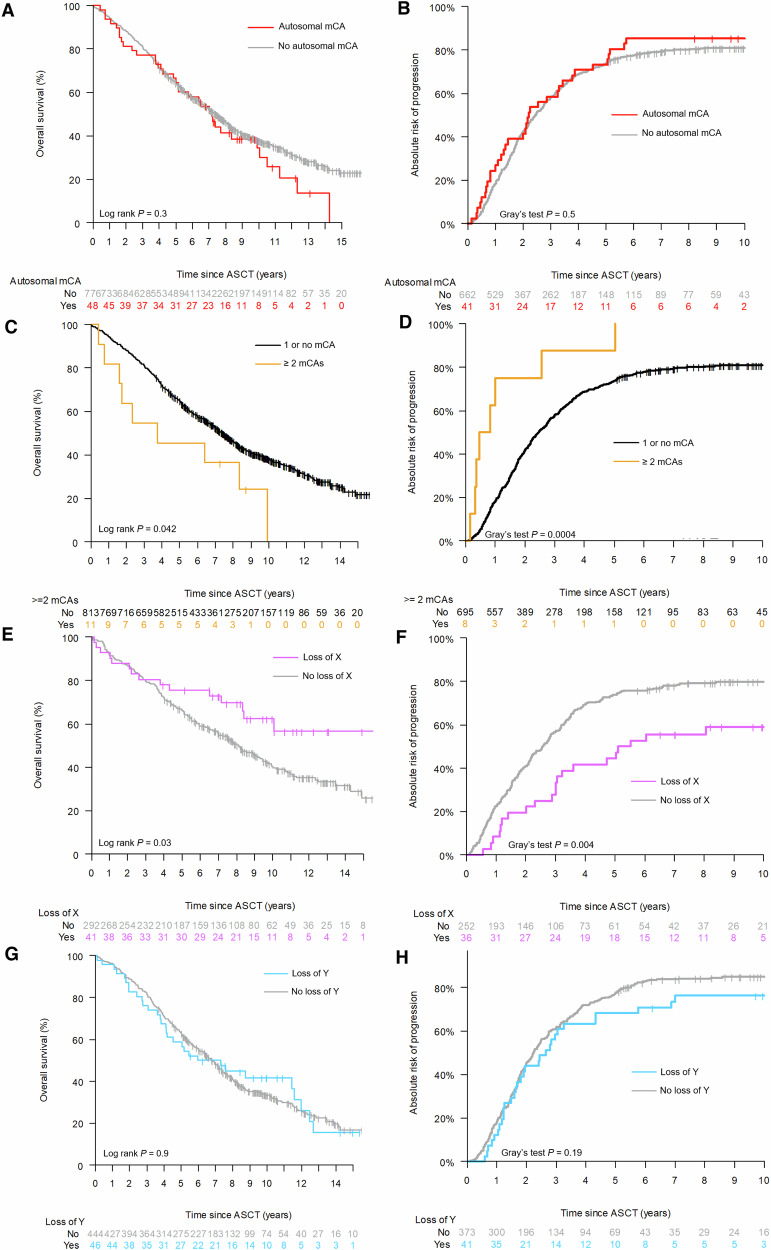
Survival in patients with mCAs. **A** Overall survival among patients with autosomal mCA versus those without. **B** Cumulative incidence of myeloma progression among patients with autosomal mCA versus those without. **C** Overall survival among patients with two or more mCAs. **D** Cumulative incidence of myeloma progression among patients with two or more mCAs. **E** Overall survival among female patients with loss of chromosome X versus those without. **F** Cumulative incidence of myeloma progression among female patients with loss of chromosome X versus those without. **G** Overall survival among male patients with loss of chromosome Y versus those without. **H** Cumulative incidence of myeloma progression among male patients with loss of chromosome Y versus those without.

**Fig. 5:**
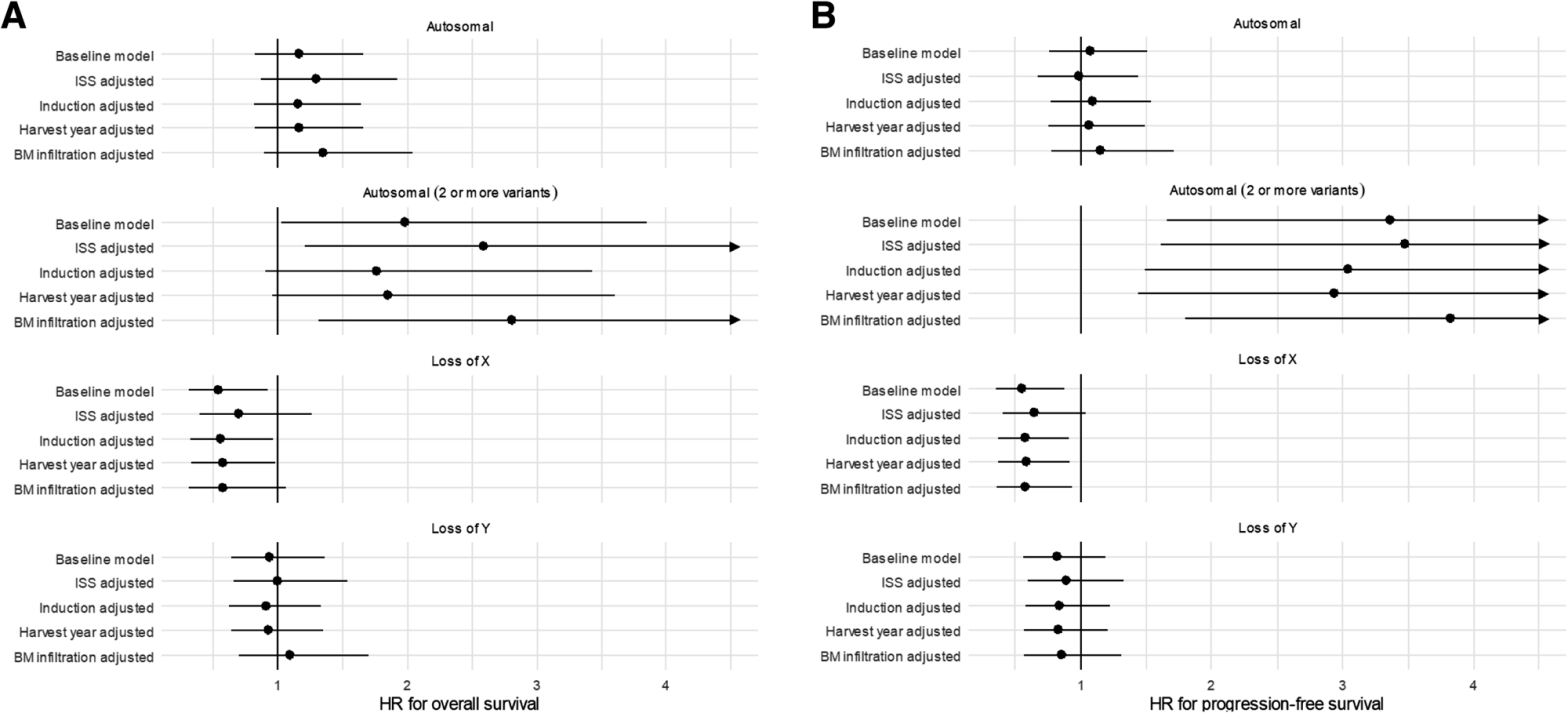
Multivariate analyses of impact of mCAs on survival. **A** Results of multivariate Cox models for overall survival. **B** Results of multivariate cause-specific Cox models for progression-free survival. Multivariate Cox models adjusted for ISS (International Staging System), type of induction therapy, time period of stem cell harvest /ASCT, and BM (bone marrow) myeloma infiltration at diagnosis. Progression-free survival models are cause-specific. All models are adjusted for age.

The median follow-up time was 6.4 years. Median overall survival was 7.2 (95% CI 5.1–10.5) years in carriers of autosomal mCAs and 7.4 (95% CI 6.8–8.1, Fig. 4A) in those without autosomal mCAs. In an age-adjusted Cox regression model, presence of one or more autosomal mCAs was not associated with different overall survival after ASCT (HR 1.17, *p* = 0.38, Fig. 5A). Sensitivity analyses with additional adjustments for either ISS, type of induction, year of stem cell harvest, or bone marrow (BM) myeloma infiltration at diagnosis did not alter this finding (Supplementary Table 8A). Similarly, autosomal mCAs were not associated with altered cumulative incidence (Fig. 4B) or cause-specific hazards of progression (Fig. 5B).

Patients who had ≥2 autosomal mCAs had a median overall survival of 3.8 years (95% CI 1.7–inf) compared to 7.4 (6.9–8.0 Fig. 4C). In an age-adjusted Cox regression model, the HR for all-cause death was 2.0 (*p* = 0.04, Fig. 5A) among carriers of two or more autosomal mCAs. HR estimates ranged from 1.9 to 2.8 in sensitivity analyses (Supplementary Table 8A). Statistical significance of multiple autosomal mCAs for inferior overall survival was noted in the models adjusted for age, ISS stage, and bone marrow infiltration. However, in models with adjustment for type of induction regimen and stem cell harvest year, effect estimates did not reach statistical significance. With regards to myeloma progression, ≥2 autosomal mCAs were associated with increased risk of progression in all models (Figs. 4D and 5B; multivariate progression-specific hazards ratios ranging from 3.0 to 3.9, Supplementary Table 8B).

Contrary to this, loss of chromosome X in females was significantly associated with superior overall survival: Median survival was 8.1 years (95% CI 7.0–9.4 years) among those without loss of X, while the median was not reached among those with loss of X (Fig. 4E). Loss of the X chromosome was associated with a lower risk of death in a Cox model adjusted for age (HR 0.54, *p* = 0.03, Fig. 5A) and with sensitivity analysis hazards ratios ranging from 0.57 to 0.70 depending on model. We also observed a decreased incidence of myeloma progression in females with loss of chromosome X (Fig. 4F, Gray’s test *p* = 0.004), which also was evident in multivariate cause-specific Cox models with HR estimates ranging between 0.55 and 0.65 and *p* values between 0.01 and 0.07 (Fig. 5B).

Males with loss of the Y chromosome did not have a different overall survival (Fig. 4G, age-adjusted *p* = 0.77) compared with males with chromosome Y. No effect of loss of Y was seen in multivariate cause-specific Cox models for overall survival (Fig. 5A). Loss of chromosome Y was not associated with altered rates of myeloma progression (Fig. 4H) and did not have an effect in multivariate cause-specific Cox models of progression-free survival (Fig. 5B).

### Detection of myeloma chromosomal alterations in the stem cell harvest product

Previous studies using flow cytometry have shown that aberrant plasma cells can be identified in stem cell harvest products and are associated with inferior outcomes [19–21]. It is unknown whether these plasma cells are truly malignant myeloma clones. The increased risk of both all-cause death and myeloma progression in patients with multiple autosomal mCAs lead us to investigate if these somatic chromosomal alterations in the stem cell harvest product was of myeloma origin. Of the 979 patients analyzed for mCAs, we had data from 407 patients on occurrence of common myeloma chromosomal alterations detected by FISH in bone marrow sorted CD138+ cells, i.e., 1q amplification, 11q amplification, 13q loss, and 17p loss, as reported to the DaMyDa registry. In only one patient we identified a concurrent mCA and FISH aberration occurring on the same chromosome (Supplementary Table 9A). This patient had by FISH analysis of CD138+ myeloma cells detected an amplification of chromosome 1q, while chromosome 1q CN-LOH was barely detectable by mCA analyses of the stem cell harvest product with an estimated cell fraction of 1.1% (lower cut-off for detection of mCAs is 1.0%).

To further investigate possible contamination of myeloma cells we specifically analyzed the five patients who had an excessive high number of autosomal mCAs (≥4 mCAs). In all five patients, the identified mCAs were clonal, i.e., different mCAs having similar cell fraction and were in genomic areas known to be disrupted in myeloma cells (Supplementary Fig. A) [18, 22]. Of note, all five patients were treated prior to 2008 and thus with less effective induction regimens without bortezomib or lenalidomide. We were able to retrieve bone marrow myeloma FISH analysis on one of the five patients (not reported to the DaMyDa registry, and thus not identified the initial comparison above), which interestingly showed concurrent mCA and FISH abnormalities for chr6q, chr13q, chr14q, and chr17p (Supplementary Table 9B). In summary, this strongly indicates that clonal aberrations of myeloma cells previously have contaminated the stem cell harvest products and that minimal residual disease can be detected using SNP arrays with this novel bioinformatic method.

**Fig. 6:**
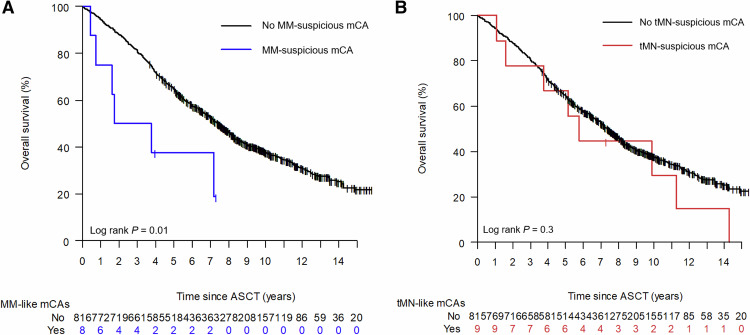
Suspected clonal origin and outcome. **A** Overall survival according to chromosomal aberrations associated with multiple myeloma (at least one of the following; amplification of chromosome 1, loss of chromosome 6q, 13, 14q, 16q, or 17p). **B** Overall survival according to mCAs associated with therapy-related leukemia (at least one of the following; loss of chromosome 5, 7, 12, or 17p).

Additionally, we isolated identified mCA variants that were known as common MM genetic aberrations (either amplification of chromosome 1, or loss of chromosome 6q, 13q, 14q, 16q, or 17p) [18]. Patients with these myeloma-suspicious mCAs (*n* = 8) had 5-year overall survival of 37.5% compared to 64.9% in the remaining patients (Fig. 6A, HR 2.7, unadjusted *p* = 0.017). We also investigated mCAs known to be associated with therapy-related leukemia [23] (loss of chromosome 5, 7, 12p, 17p; *n* = 12), but these were not associated with inferior overall survival (Fig. 6B, unadjusted *p* = 0.3).

In a separate analysis, we also investigated whether exclusion of the patients with suspected MM contaminants in their harvest product (the genetic aberrations mentioned above), would alter the findings for patients with ≥2 autosomal mCAs. With caution for very small numbers, it seems as if multiple mCAs do not infer worse outcomes with regards to overall survival and myeloma progression, when patients with possible myeloma contaminants are excluded (Supplementary Fig. 4A, B).

## Discussion

The age-related acquisition of chromosomal abnormalities in hematopoietic cells, i.e., mCAs, has been shown to impact healthy individuals, leading to impaired survival and increased rates of infections [2, 3, 24]. By studying a cohort of well-characterized myeloma patients undergoing ASCT with extensive long-term follow-up, we describe the landscape of mCAs in multiple myeloma and their effect on clinical outcomes.

As expected, the incidence of mCAs was highly age-related in patients with myeloma and 20% of patients between 60 and 70 years had a detectable mCA. This is more frequent than what has been reported in healthy individuals, with a prevalence of approximately 14% in same age-group in the UK Biobank [2] and in the Japanese Biobank [24, 25]. Both biobank studies found that occurrence of an mCA was associated with an increased risk of developing blood cancers (including multiple myeloma), which can explain why we find a higher prevalence in the myeloma cohort. Likewise, loss of chromosome X was more frequent in patients with myeloma (12%) than what has been reported for healthy individuals in the UK biobank (~5% in the same age range). However, it should be stressed that the detection rate of mCAs is cohort-dependent due to use of different genetic arrays used and enhanced quality of phasing with increasing sample numbers, so direct comparison should be done with caution. We did not observe differences in frequency of mCAs between patients treated with different induction regimens or drug classes (bortezomib, cyclophosphamide, thalidomide/lenalidomide) and expect that the identified mCAs are unrelated to myeloma therapy given prior to ASCT.

Patients with sex chromosome losses had significantly lower myeloma infiltration in their bone marrow at diagnosis. We speculate that HSC’s with loss of a sex chromosome may be more resilient than wildtype HSC’s, when stressed by proliferating neoplastic plasma cells, thus leading to lower infiltration of myeloma in the bone marrow.

With regards to events after ASCT, there were strikingly better outcomes for female patients with clonal loss of the X chromosome. Compared to females without loss of X, they had significantly higher rates of post-transplant CR/VGPR, longer overall survival, lower risk of myeloma progression after ASCT. The reasons for these substantial differences are not clear. Loss of X in the malignant myeloma clones has previously been examined and is not correlated with inferior survival [22, 26].

Overexpression of cancer-testis antigens (CTAs; located on the X chromosome) have in multiple studies been associated with inferior survival in MM [27, 28]. We speculate that the myeloma clone in patients with hematopoietic loss of X may also have lost the X chromosome and thus have lower expression of CTAs. This would potentially lead to a less aggressive form of multiple myeloma and explain the superior outcomes associated with loss of X. Further studies on CD138^+^-isolated myeloma cells from patients with loss of X are needed to confirm this. Additionally, the steroid sulfatase gene (*STS*), which is essential for estrogen synthesis, is located on the X chromosome [29]. Estrogens have been shown to promote development of multiple myeloma [30], while antiestrogens have been shown to induce myeloma apoptosis [31]. Thus, losing the *STS* gene in hematopoietic cells, may decrease the production of estrogen in the bone marrow and thus slow the progression of myeloma. It should be mentioned that the current method to detect mCAs does not provide information on X chromosome inactivation. Additionally, we did not observe survival differences for male patients with myeloma who had lost the Y chromosome. This is in contrast to several reports in healthy and diseased individuals, where loss of chromosome Y is associated with an increased all-cause mortality and cancer-related mortality [32, 33]. However, these studies used a different strategy to detect loss of chromosome Y which is restricted to higher cell fractions, so it is not directly comparable to our analysis with detection of many low-frequent losses of Y.

Patients with myeloma harboring autosomal mCAs had a borderline significant increased risk of hospital admission for infection. This effect has also been observed in a large study of healthy individuals [3]. In larger cohorts, hemoglobin level has been found to be marginally lower in individuals with autosomal mCAs [24]. We found numerically lower hemoglobin levels in myeloma patients with mCAs, but this was not significant when adjusted for confounders.

The findings of this study should be interpreted with caution due to the larger number of tests performed. We cannot exclude that the association between loss of chromosome X and better outcomes is a chance finding. Therefore, this result should be validated before clinical use.

A total of 12 patients in the cohort (1.2%) had multiple autosomal mCAs detected in their stem cell harvest material, which was found to correlate with poor outcomes. When analyzing these multiple mCAs more closely, we found that the chromosomal alterations in patients with multiple mCAs were almost all known from cytogenetic studies of malignant myeloma cells [18]. Our suspicion of myeloma contamination in the stem cell harvest product was confirmed in one patient with available diagnostic CD138^+^ FISH data. There was a large overlap of chromosomal changes found by array-based mCA analysis and by FISH analysis, supporting that myeloma cells were present in the autologous harvest product. We furthermore noticed that all patients with a high number of mCAs (>3) were treated prior to 2012, and thus with less effective induction regimens (and poorer depths of response pre-transplant), than those commonly used today. When excluding patients with suspected myeloma contaminants, there was seemingly no poor prognostic effect of multiple autosomal mCAs, underpinning that autosomal mCA in the myeloma setting is not of prognostic significance, while remaining myeloma cells (i.e., MRD) are. This is in agreement with recent data on the lacking prognostic effect of clonal hematopoiesis in patients with MM [4].

In conclusion, in this large cohort with long-term follow-up, female multiple myeloma patients with loss of the X chromosome had better post-transplant response, lower risk of myeloma progression, and longer overall survival, despite being older. Multiple autosomal mCAs were associated with inferior survival and increased progression, but is driven by detection of circulating myeloma cells. It was possible to detect bone fide residual myeloma cells in the stem cell product with this novel bioinformatic analysis of standard SNP arrays. Further research on the biological implications of loss of the X chromosome is warranted.

## Supplementary information

Supplementary Tables and figures

Pile-up plot of genomic locations of mCAs found in the cohort

Data sheet with Cox models of overall survival

Data sheet with Cox models of progression

Supplemental Note 1

## Data Availability

The clinical and genetic datasets are regulated by the European Union General Data Protection Regulation law. Contact the corresponding author for access to data.
