# Supplementary material for: Mosaic chromosomal alterations in hematopoietic cells and clinical outcomes in patients with multiple myeloma
Source: Leukemia. 2024 Sep 2;38(11):2456–65. doi: 10.1038/s41375-024-02396-3 (PMC11518982; doi:10.1038/s41375-024-02396-3)

Mosaic chromosomal alterations in hematopoietic cells and clinical outcomes in patients with multiple myeloma

Simon Husby*[1,2], Morten Tulstrup*[1,2], Mads Harsløf[1,2], Christian Nielsen[3,4], Eva Haastrup[5], Lene Hyldahl Ebbesen[6], Mette Klarskov Andersen[7], Maroulio Pertesi[8], Christian Brieghel[1], Carsten U. Niemann[1], Björn Nilsson[8], Agoston Gyula Szabo[1], Niels Frost Andersen[6], Niels Abildgaard[9], Annette Vangsted*[1,10], Kirsten Grønbæk*[1,2,10]

Supplemental material

Supplemental Table 1: Frequency of autosomal and sex chromosome variants in the cohort

Variables	N pts	Frequency
No autosomal	916	0.939
Autosomal	60	0.061
No X loss (female)	350	0.873
X loss (female)	51	0.127
No Y loss (male)	519	0.904
Y loss (male)	55	0.096

Supplemental Table 2: Sizes of mCA variants

Size category (Mb)	N mCA variants	Frequency
(0,1]	12	0.114
(1,10]	9	0.086
(10,50]	34	0.324
(50,100]	28	0.267
(100,Inf]	22	0.210

Supplemental Table 3: Baseline characteristics with subgroups

		All patients			Female patients			Male patients		
		No autosomal mCA	Autosomal mCA	p	No loss of X	Loss of X	p	No loss of Y	Loss of Y	p
No. of patients		916	60		350	51		519	55	
Age		58.8 (7.6)	61.1 (6.0)	0.034	58.1 (7.9)	61.5 (7.6)	0.012	58.8 (7.2)	62.6 (5.3)	0.001
Sex (%)	Female	376 (41.1)	25 (41.7)	1.000	350 (100.0)	51 (100.0)		0 (0.0)	0 (0.0)	
	Male	539 (58.9)	35 (58.3)		0 (0.0)	0 (0.0)		519 (100.0)	55 (100.0)	
Type of M protein	IgA	143 (21.7)	9 (23.7)	0.965	59 (24.4)	8 (23.5)	0.106	79 (20.6)	6 (16.7)	0.929
	IgG	403 (61.2)	23 (60.5)		148 (61.2)	17 (50.0)		238 (62.0)	23 (63.9)	
	Light-chain	66 (10.0)	3 (7.9)		23 (9.5)	8 (23.5)		34 (8.9)	4 (11.1)	
	Other	46 (7.0)	3 (7.9)		12 (5.0)	1 (2.9)		33 (8.6)	3 (8.3)	
Hemoglobin [mmol/L]		6.8 (1.2)	6.6 (1.2)	0.339	6.5 (1.0)	6.8 (1.2)	0.159	6.9 (1.4)	7.3 (1.1)	0.059
β2M [mg/L]		4.9 (4.9)	6.4 (8.7)	0.082	5.3 (5.8)	4.7 (4.4)	0.561	5.0 (5.1)	3.8 (3.2)	0.153
Albumin [g/L]		36.0 (6.9)	37.1 (6.0)	0.294	36.3 (6.5)	37.2 (7.2)	0.444	35.8 (7.2)	35.9 (5.5)	0.936
LDH [U/L]		191.2 (98.8)	186.6 (103.9)	0.762	197.2 (99.1)	190.5 (128.5)	0.713	187.6 (98.9)	184.2 (71.1)	0.826
Stage (ISS)	1	202 (36.1)	14 (37.8)	0.952	74 (34.6)	12 (38.7)	0.843	114 (36.2)	16 (44.4)	0.522
	2	211 (37.7)	13 (35.1)		82 (38.3)	12 (38.7)		116 (36.8)	13 (36.1)	
	3	147 (26.2)	10 (27.0)		58 (27.1)	7 (22.6)		85 (27.0)	7 (19.4)	
Bone marrow infiltration (%)		42.1 (24.0)	35.9 (20.4)	0.109	42.1 (23.4)	32.5 (21.5)	0.026	43.3 (24.2)	31.2 (20.2)	0.004
M protein [g/L]		35.1 (24.2)	28.5 (24.9)	0.155	33.9 (23.6)	27.8 (28.5)	0.219	35.8 (24.5)	37.1 (22.3)	0.795
Days from induction to ASCT		148.5 (186.6)	145.8 (121.9)	0.928	133.6 (102.8)	160.8 (288.8)	0.277	155.7 (213.2)	161.3 (159.5)	0.872
Induction regimen	CyDex	262 (33.3)	17 (35.4)	0.905	105 (35.2)	9 (22.0)	0.256	146 (32.5)	19 (41.3)	0.385
	Other	63 (8.0)	5 (10.4)		26 (8.7)	7 (17.1)		30 (6.7)	5 (10.9)	
	VAD	66 (8.4)	3 (6.2)		26 (8.7)	3 (7.3)		38 (8.5)	2 (4.3)	
	VCd	195 (24.8)	9 (18.8)		71 (23.8)	14 (34.1)		111 (24.7)	7 (15.2)	
	Vd	154 (19.6)	11 (22.9)		56 (18.8)	7 (17.1)		91 (20.3)	11 (23.9)	
	VimidD	47 (6.0)	3 (6.2)		14 (4.7)	1 (2.4)		33 (7.3)	2 (4.3)	

Data are presented as means with standard deviation in parenthesis, unless otherwise noted. Of the 976 patients analyzed for mosaic chromosomal alterations, there was clinical annotation on 825 patients. Missing data on categorical variables is shown in the figure. For the numerical variables (hemoglobin, β2M, albumin, LDH, bone marrow infiltration, M protein level) there were missing data on 311, 311, 254, 241, 309, and 404 patients, respectively. With regards to analysis of sex, the SNP array data were used to impute sex (this classified the sex 100% correctly in the patients annotated with information om female/male). Eight patients had both an autosomal mCA and loss of a sex chromosome and are thus included in two columns. Therefore, the total fraction of patients from the full cohort represented in this table surpasses 100%. IgA, immunoglobulin A; IgG, immunoglobulin G; Other, immunoglobulin E, D or not-determined; β2M; beta-2-microglobulin; LDH, lactate dehydrogenase; ISS, International Staging System; ASCT, autologous stem cell transplant. Peripheral blood biochemistry data (hemoglobin, beta-2-microglobulin, albumin, lactate dehydrogenase, M protein) and bone marrow infiltration is from time of diagnosis.

Supplemental Table 4: Logistic regression of variants and myeloma bone marrow infiltration

	OR	lower 95 CI	upper 95 CI	P	Overall P value BMinf
Autosomal (N = 619)					
(Intercept)	0,934	0,804	1,085	0,373	
age_harvest	1,002	1,000	1,005	0,071	
computed_genderM	1,001	0,965	1,038	0,949	
bminf(25,50]	0,998	0,955	1,043	0,931	
bminf(50,75]	0,973	0,926	1,023	0,281	
bminf(75,100]	0,968	0,908	1,032	0,315	0,550
Autosomal >= 2 (N = 619)					
(Intercept)	0,952	0,888	1,021	0,170	
age_harvest	1,001	1,000	1,002	0,153	
computed_genderM	0,999	0,982	1,016	0,925	
bminf(25,50]	1,009	0,989	1,030	0,366	
bminf(50,75]	1,023	1,000	1,047	0,053	
bminf(75,100]	1,032	1,002	1,064	0,036	0,094
X loss (N = 258)					
(Intercept)	0,946	0,700	1,279	0,718	
age_harvest	1,004	0,999	1,009	0,128	
bminf(25,50]	0,924	0,840	1,016	0,102	
bminf(50,75]	0,949	0,853	1,057	0,343	
bminf(75,100]	0,843	0,733	0,971	0,018	0,093
Y loss (N = 361)					
(Intercept)	0,782	0,605	1,011	0,062	
age_harvest	1,006	1,002	1,010	0,004	
bminf(25,50]	0,980	0,911	1,053	0,575	
bminf(50,75]	0,936	0,863	1,016	0,115	
bminf(75,100]	0,910	0,820	1,009	0,076	0,201
XY loss (N = 619)					
(Intercept)	0,867	0,712	1,056	0,157	
age_harvest	1,005	1,002	1,008	0,002	
computed_genderM	0,975	0,930	1,023	0,308	
bminf(25,50]	0,954	0,901	1,011	0,113	
bminf(50,75]	0,940	0,881	1,003	0,063	
bminf(75,100]	0,882	0,811	0,959	0,003	0,022

Abbreviations; bminf, bone marrow myeloma infiltration percentage; OR, odds ratio; CI, confidence interval.

Supplemental Table 5: Logistic regression of M protein and occurrence of mCAs

	OR	lower 95 CI	upper 95 CI	P	Overall P value M protein
autosomal (N = 514)					
(Intercept)	0,003	0,000	14865097,557	0,690	
mprot(16,33]	0,824	0,269	2,657	0,736	
mprot(33,51.5]	0,629	0,187	2,119	0,444	
mprot(51.5,Inf]	0,552	0,152	1,933	0,349	0,764
type_m_kompIgG	0,963	0,382	2,762	0,939	
type_m_kompOther	1,089	0,151	5,167	0,921	
computed_genderM	1,101	0,481	2,632	0,822	
age_harvest	1,028	0,468	3,440	0,955	
age_harvest_sq	1,000	0,990	1,007	0,931	
X loss (N = 207)					
(Intercept)	0,004	0,000	8589841750,321	0,774	
mprot(16,33]	0,302	0,073	1,200	0,087	
mprot(33,51.5]	0,342	0,075	1,421	0,143	
mprot(51.5,Inf]	0,590	0,140	2,443	0,461	0,582
type_m_kompIgG	0,609	0,212	1,842	0,362	
type_m_kompOther	0,568	0,026	4,625	0,640	
age_harvest	1,014	0,373	5,609	0,983	
age_harvest_sq	1,001	0,987	1,010	0,867	
Y loss (N = 307)					
(Intercept)	0,000	0,000	301,436	0,199	
mprot(16,33]	1,336	0,308	6,838	0,704	
mprot(33,51.5]	2,146	0,594	10,176	0,276	
mprot(51.5,Inf]	1,875	0,508	8,957	0,374	0,650
type_m_kompIgG	0,947	0,350	3,016	0,920	
type_m_kompOther	1,542	0,288	7,090	0,584	
age_harvest	2,664	0,673	23,196	0,277	
age_harvest_sq	0,993	0,975	1,004	0,320	
XY loss (N = 514)					
(Intercept)	0,000	0,000	826,038	0,251	
mprot(16,33]	0,665	0,250	1,792	0,411	
mprot(33,51.5]	0,962	0,386	2,495	0,934	
mprot(51.5,Inf]	1,064	0,426	2,762	0,896	0,813
type_m_kompIgG	0,772	0,381	1,649	0,484	
type_m_kompOther	1,180	0,303	3,833	0,794	
computed_genderM	0,834	0,442	1,590	0,575	
age_harvest	1,590	0,663	5,779	0,400	
age_harvest_sq	0,997	0,987	1,004	0,510	

Abbreviations; OR, odds ratio; CI, confidence interval; Inf, infinite; mprot, M protein; type_m_kompIgG, M protein IgG; type_m_kompOther, Non-IgG M protein; computed_genderM, male gender.

Supplemental Table 6: Logistic regression of induction therapies and occurrence of mCAs

A)

	OR	lower 95	upper 95	P
**Autosomal (N = 825)**				
(Intercept)	0,009	0,001	0,095	0,000
Other	1,259	0,401	3,333	0,663
VAD	2,226	0,816	5,540	0,097
VCd	1,092	0,516	2,273	0,815
Vd	1,144	0,518	2,449	0,732
VimidD	1,272	0,353	3,632	0,678
age_harvest	1,034	0,995	1,078	0,105
**Autosomal (N = 825)**				
(Intercept)	0,015	0,001	0,136	0,000
bortezomib	1,008	0,589	1,730	0,978
age_harvest	1,028	0,990	1,071	0,166
**Autosomal (N = 825)**				
(Intercept)	0,017	0,001	0,155	0,001
cyclophosphamide	0,754	0,443	1,293	0,300
age_harvest	1,029	0,991	1,071	0,149
**Autosomal (N = 825)**				
(Intercept)	0,015	0,001	0,137	0,000
imid	1,059	0,311	2,727	0,915
age_harvest	1,028	0,990	1,070	0,161

B)

	OR	lower 95 CI	upper 95 CI	P
**X loss (N = 333)**				
(Intercept)	0,002	0,000	0,042	0,000
Other	3,170	1,033	9,458	0,038
VAD	1,896	0,391	7,160	0,373
VCd	2,095	0,859	5,332	0,108
Vd	1,298	0,439	3,702	0,626
VimidD	0,824	0,043	4,958	0,860
age_harvest	1,063	1,014	1,121	0,016
**X loss (N = 333)**				
(Intercept)	0,004	0,000	0,061	0,000
bortezomib	1,203	0,615	2,378	0,589
age_harvest	1,061	1,012	1,120	0,020
**X loss (N = 333)**				
(Intercept)	0,004	0,000	0,066	0,000
cyclophosphamide	0,826	0,424	1,626	0,575
age_harvest	1,065	1,015	1,123	0,014
**X loss (N = 333)**				
(Intercept)	0,004	0,000	0,063	0,000
imid	0,465	0,025	2,419	0,466
age_harvest	1,063	1,015	1,121	0,015

C)

	OR	lower 95 CI	upper 95 CI	P
**Y loss (N = 491)**				
(Intercept)	0,000	0,000	0,007	0,000
Other	1,480	0,451	4,171	0,481
VAD	0,693	0,105	2,667	0,640
VCd	0,425	0,159	1,020	0,067
Vd	0,795	0,344	1,753	0,576
VimidD	0,384	0,059	1,442	0,218
age_harvest	1,111	1,050	1,186	0,001
**Y loss (N = 491)**				
(Intercept)	0,000	0,000	0,006	0,000
bortezomib	0,542	0,284	1,019	0,059
age_harvest	1,114	1,053	1,188	0,000
**Y loss (N = 491)**				
(Intercept)	0,000	0,000	0,009	0,000
cyclophosphamide	0,787	0,423	1,480	0,450
age_harvest	1,102	1,044	1,171	0,001
**Y loss (N = 491)**				
(Intercept)	0,000	0,000	0,008	0,000
imid	0,777	0,180	2,327	0,690
age_harvest	1,102	1,044	1,171	0,001

Abbreviations; OR, odds ratio; CI, confidence interval; VCd, velcade-cyclophosphamide-dexamethasone, VAD, vincristine-doxorubicin-dexamethasone; Vd, velcade-dexamethasone; VimidD, velcade-thalidomide/lenalidomide-dexamethasone; imid, thalidomide/lenalidomide.

Supplemental Table 7: High-risk multiple myeloma cytogenetics and occurrence of mCA

	Autosomal mCA				Loss of X				Loss of Y	
**HR MM cytogenetics**	No	Yes		**HR MM cytogenetics**	No	Yes		**HR MM cytogenetics**	No	Yes
No	221	14		No	86	8		No	129	11
Yes	125	6		Yes	43	10		Yes	72	6
	Chi-square p = 0.35				Chi-square p = 0.07				Chi-square p = 0.41	

Supplemental Figure 1: Distribution of clonal fraction with regards to type of mCA

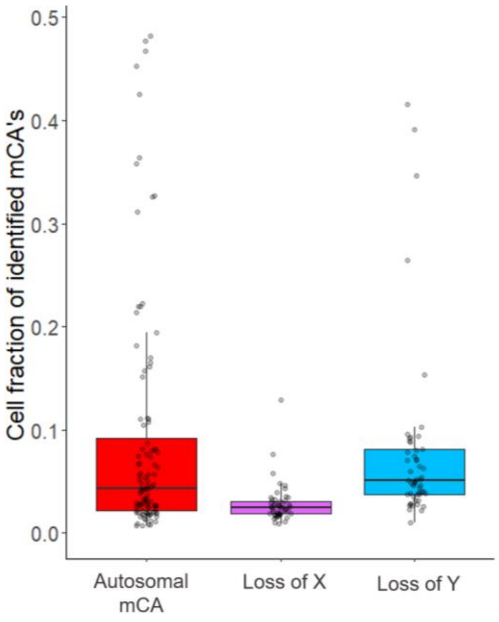

Supplemental Figure 2: Days admitted to hospital (per days alive) in first year after ASCT

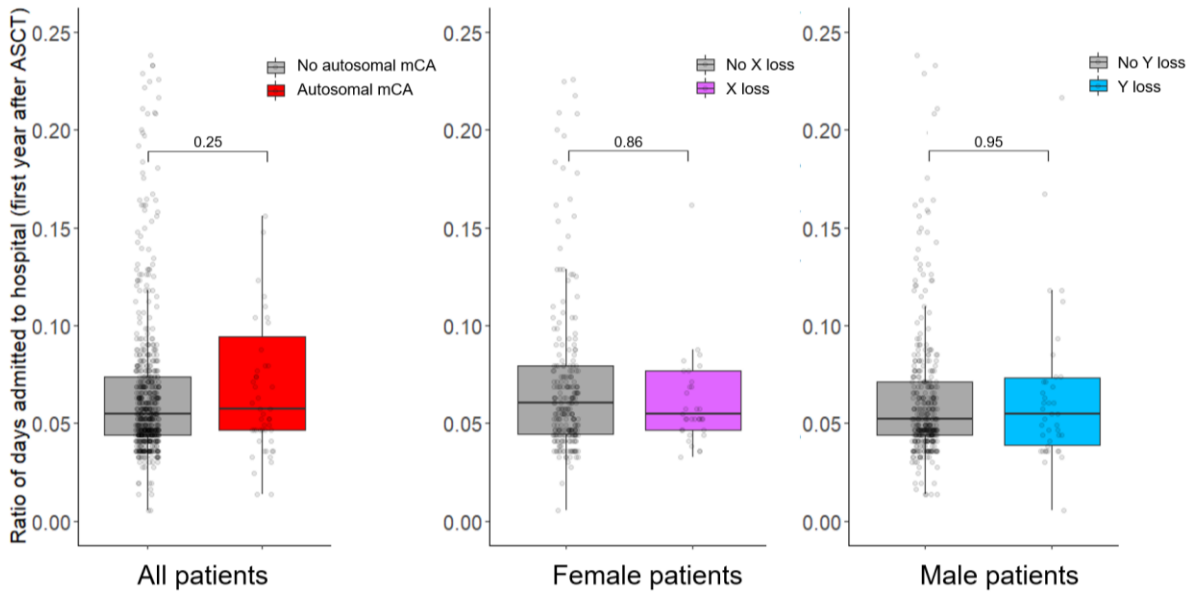

Supplemental Figure 3: Days admitted to hospital (per days alive) in year 2-5 after ASCT

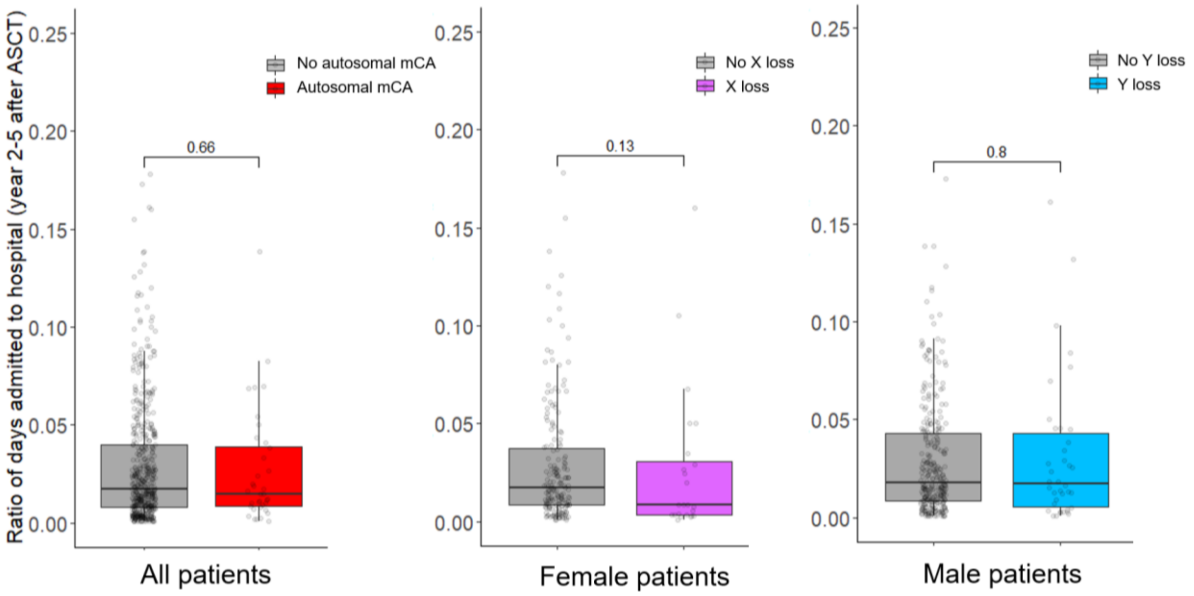

Supplemental Table 8 A-B: Cox models of overall survival and cause-specific hazards of progression.

All Cox models are attached in the full excel-files ‘OS_cox.excl’ and ‘progression_cox.excl’.

Regarding the sensitivity analyses of ISS score as a time-dependent covariate: An ISS score of 3 violated the proportional hazards assumption and was therefore split at 0-2 years and 2-infinite years after ASCT (abbreviated ‘tt’). In progression analyses, the PH assumption was violated for the harvest year category 2012-2017, and we added an additional parameter varying with log(time) to accommodate this.

Abbreviations to the attached file; HR, harzard ratio; CI, confidence interval; BMinf, bone marrow infiltration; ISS, International Staging System; VCd, velcade-cyclophosphamide-dexamethasone, VAD, vincristine-doxorubicin-dexamethasone; Vd, velcade-dexamethasone; VimidD, velcade-thalidomide/lenalidomide-dexamethasone; imid, thalidomide/lenalidomide.

Supplemental Figure A: Patients with >3 mCAs

Pt. A

computed_gender	chrom	beg_grch37	end_grch37	length	p_arm	q_arm	type	cf	chr_group	size_Mb	size_cat
M	1	171080615	249250621	78170006	N	T	Undetermined	0.0304	autosomal	78.17001	(50,100]
M	3	0	198022430	198022430	T	T	Gain	0.0298	autosomal	198.02243	(100,Inf]
M	9	0	141213431	141213431	T	T	Gain	0.0305	autosomal	141.21343	(100,Inf]
M	17	0	81195210	81195210	T	T	Loss	0.0219	autosomal	81.19521	(50,100]

Chrom, chromosome; beg_/end_, coordinates on human genome reference consortium build 37; cf, cell fraction; chr_group, group of chromosomal alteration (i.e. autosomal or sex chromosome); size_Mb, size of mCA in megabases; size_cat, categorized size of mCA (1-10, 10-50, 50-100,100-∞ megabases); F, female; M, male.

Comment:

Similar clone size (3% cell fraction). 1q amplification, chr3 amplification, chr9 amplification and chr17 loss are all known genetic aberrations in multiple myeloma cells.

Pt. B

computed_gender	chrom	beg_grch37	end_grch37	length	p_arm	q_arm	type	cf	chr_group	size_Mb	size_cat
M	1	150870629	213999434	63128805	N	Y	Gain	0.0270	autosomal	63.12880	(50,100]
M	1	225610492	249250621	23640129	N	T	Gain	0.0541	autosomal	23.64013	(10,50]
M	10	0	37505159	37505159	T	N	Undetermined	0.0197	autosomal	37.50516	(10,50]
M	13	37217459	115169878	77952419	N	T	CN-LOH	0.0181	autosomal	77.95242	(50,100]
M	22	16000000	51304566	35304566	N	T	Undetermined	0.0267	autosomal	35.30457	(10,50]

Comment:

Similar clone size (~2% cell fraction). Chr1q amplification, chr13 loss, and chr22 amplification are known genetic aberrations in multiple myeloma cells.

Pt. C

computed_gender	chrom	beg_grch37	end_grch37	length	p_arm	q_arm	type	cf	chr_group	size_Mb	size_cat
F	3	0.0e+00	198022430	198022430	T	T	CN-LOH	0.0212	autosomal	198.02243	(100,Inf]
F	4	0.0e+00	191154276	191154276	T	T	CN-LOH	0.0200	autosomal	191.15428	(100,Inf]
F	6	0.0e+00	39119837	39119837	T	N	CN-LOH	0.0196	autosomal	39.11984	(10,50]
F	9	0.0e+00	122154649	122154649	T	Y	Loss	0.0367	autosomal	122.15465	(100,Inf]
F	11	0.0e+00	135006516	135006516	T	T	Gain	0.0409	autosomal	135.00652	(100,Inf]
F	14	1.9e+07	107349540	88349540	N	T	CN-LOH	0.0406	autosomal	88.34954	(50,100]
F	17	0.0e+00	81195210	81195210	T	T	CN-LOH	0.0197	autosomal	81.19521	(50,100]

Comment:

Estimated two clones (respectively ~4% and ~2% cell fraction). Chr3 loss, chr11 amplification, chr14q loss, and chr17p loss are known genetic aberrations in multiple myeloma cells.

Supplemental Figure A: Patients with >3 mCAs (continued)

Pt. D

computed_gender	chrom	beg_grch37	end_grch37	length	p_arm	q_arm	type	cf	chr_group	size_Mb	size_cat
M	6	63951293	171115067	107163774	N	T	Loss	0.0474	autosomal	107.16377	(100,Inf]
M	8	0	146364022	146364022	T	T	Loss	0.0424	autosomal	146.36402	(100,Inf]
M	11	0	135006516	135006516	T	T	CN-LOH	0.0274	autosomal	135.00652	(100,Inf]
M	13	19000000	34654918	15654918	N	Y	Loss	0.0327	autosomal	15.65492	(10,50]
M	13	62248465	115169878	52921413	N	T	Loss	0.0461	autosomal	52.92141	(50,100]
M	14	19000000	107349540	88349540	N	T	CN-LOH	0.0436	autosomal	88.34954	(50,100]
M	16	57664233	90354753	32690520	N	T	CN-LOH	0.0174	autosomal	32.69052	(10,50]
M	17	0	20751144	20751144	T	N	CN-LOH	0.0237	autosomal	20.75114	(10,50]

Comment:

Estimated two clones (respectively ~4% and ~2% cell fraction). Chr6q loss, chr8 loss, chr13q loss, chr14q loss, chr16q loss, and chr17p loss are known genetic aberrations in multiple myeloma cells.

Pt. E

computed_gender	chrom	beg_grch37	end_grch37	length	p_arm	q_arm	type	cf	chr_group	size_Mb	size_cat
M	1	0	121164607	121164607	T	N	Loss	0.0768	autosomal	121.164607	(100,Inf]
M	1	145472788	249250621	103777833	N	T	Gain	0.1514	autosomal	103.777833	(100,Inf]
M	3	178633207	185530290	6897083	N	Y	Loss	0.0780	autosomal	6.897083	(1,10]
M	4	57477794	90062409	32584615	N	Y	Loss	0.0871	autosomal	32.584615	(10,50]
M	4	99707614	118401164	18693550	N	Y	Loss	0.0808	autosomal	18.693550	(10,50]
M	9	40601608	141213431	100611823	Y	T	CN-LOH	0.0739	autosomal	100.611823	(100,Inf]
M	13	19000000	115169878	96169878	N	T	Loss	0.0813	autosomal	96.169878	(50,100]
M	16	46644903	90354753	43709850	N	T	Loss	0.0744	autosomal	43.709850	(10,50]
M	19	0	24231229	24231229	T	N	CN-LOH	0.0381	autosomal	24.231229	(10,50]

Comment:

Estimated three clones (respectively ~15%, ~8%, and ~3.8% cell fraction). Chr1q amplification and chr16q loss are known genetic aberrations in multiple myeloma cells. Chr3q loss and chr4q loss are not common in multiple myeloma.

Supplemental Table 9A: Concurrent FISH aberrations reported to the DaMyDa registry (total n=407 patients) and mCA variants (total n=976 patients)

	mCA 1q amp. or CN-LOH	
**FISH 1q amp.**	No	Yes
No	298	0
Yes	52	1

(351 patients with conclusive FISH report on 1q amp.)

	mCA 13q del. or CN-LOH	
**FISH 13q del.**	No	Yes
No	288	0
Yes	102	0

(390 patients with conclusive FISH report on 13q del.)

	mCA 17p del. or CN-LOH	
**FISH 17p del.**	No	Yes
No	313	0
Yes	29	0

(342 patients with conclusive FISH report on 17p del.)

Full illustration of identified mCAs (including patients without available FISH reports) can be found in Figure 1.

Supplemental Table 9B: Patient with multiple concurrent FISH and mCA aberrations

FISH result		mCA findings		
**Chromosome**	**Type**	**Chromosome**	**Type**	**Cell fraction (%)**
t(6;17)(p10;q10)	Translocation	6q	Loss	4.7
-	-	8	Loss	4.2
-	-	11	CN-LOH	2.7
13	Loss	13	Loss	4.6
14q32	Translocation	14q	CN-LOH	4.4
-	-	16q	CN-LOH	1.7
17p	Loss	17p	CN-LOH	2.4

FISH results from analysis of diagnostic bone marrow $CD138^+$ cells performed in May 2006.

Mosaic chromosomal alteration (mCA) results from analysis of stem cell harvest sample (January 2007).

Supplemental Figure 4: Overall survival and cumulative incidence of progression in patients with ≥2 autosomal mCAs, when patients with suspected myeloma contaminants are excluded.

A **B**

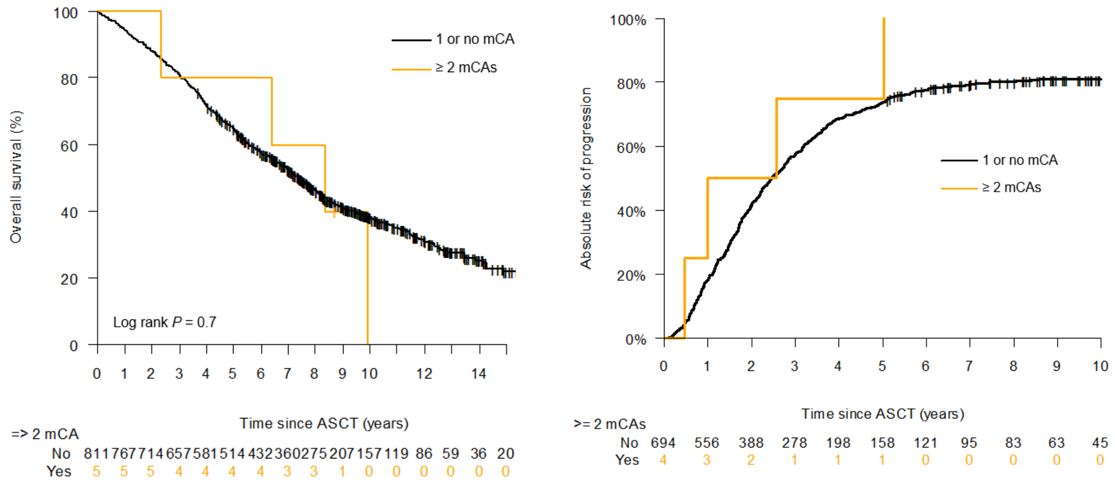

Supplement: Supplementary file 1 — Supplementary Tables and figures [file 41375_2024_2396_MOESM1_ESM.pdf]
