# Supplementary material for: Mosaic chromosomal alterations in hematopoietic cells and clinical outcomes in patients with multiple myeloma
Source: Leukemia. 2024 Sep 2;38(11):2456–65. doi: 10.1038/s41375-024-02396-3 (PMC11518982; doi:10.1038/s41375-024-02396-3)
Supplement: Supplementary file 2 — Pile-up plot of genomic locations of mCAs found in the cohort [file 41375_2024_2396_MOESM2_ESM.pdf]

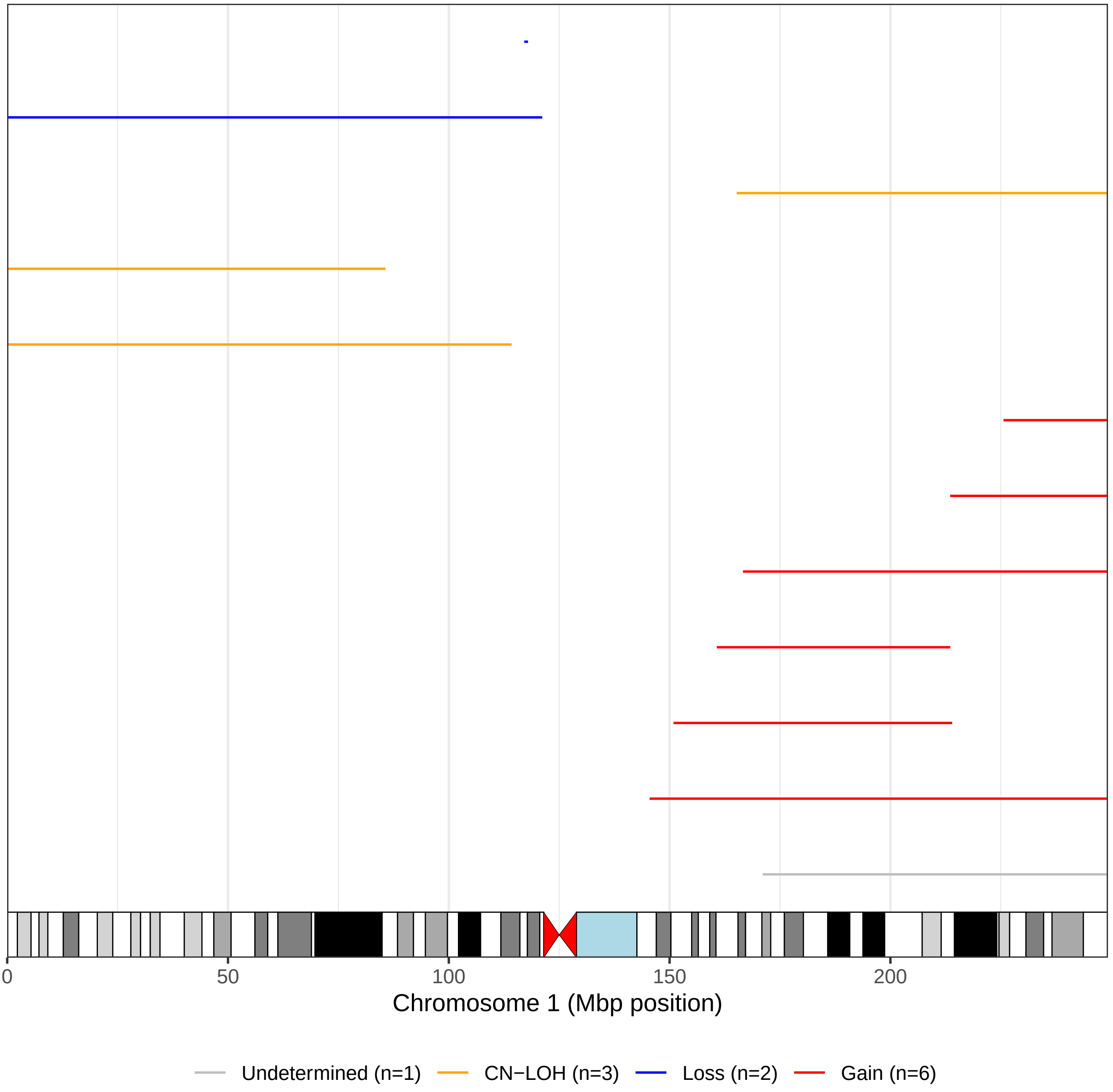

0
50
100
150
200
Chromosome 1 (Mbp position)
Undetermined (n=1)
CN−LOH (n=3)
Loss (n=2)
Gain (n=6)

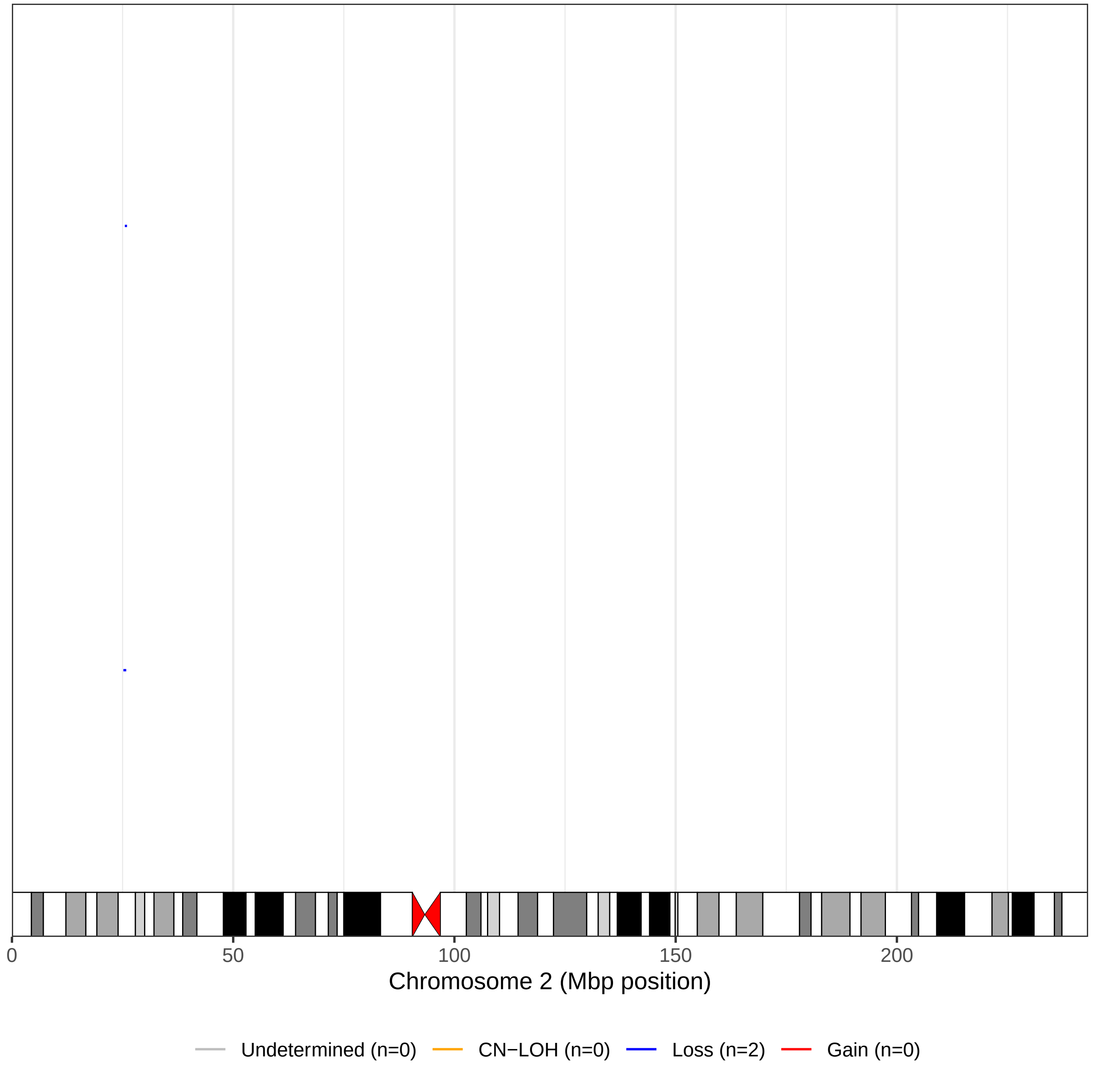
0
50
100
150
200
Chromosome 2 (Mbp position)
Undetermined (n=0)
CN−LOH (n=0)
Loss (n=2)
Gain (n=0)

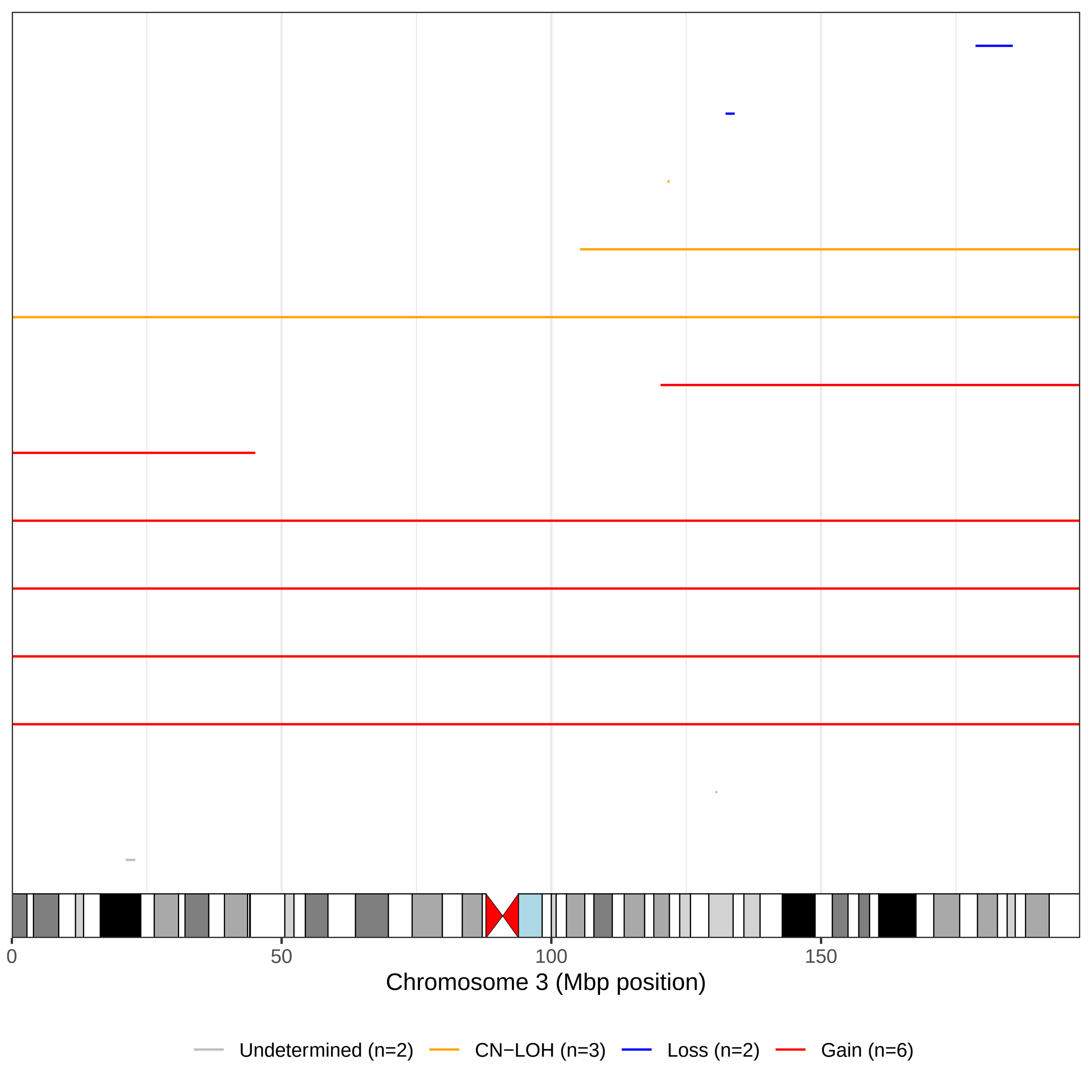

0
50
100
150
Chromosome 3 (Mbp position)
Undetermined (n=2)
CN−LOH (n=3)
Loss (n=2)
Gain (n=6)

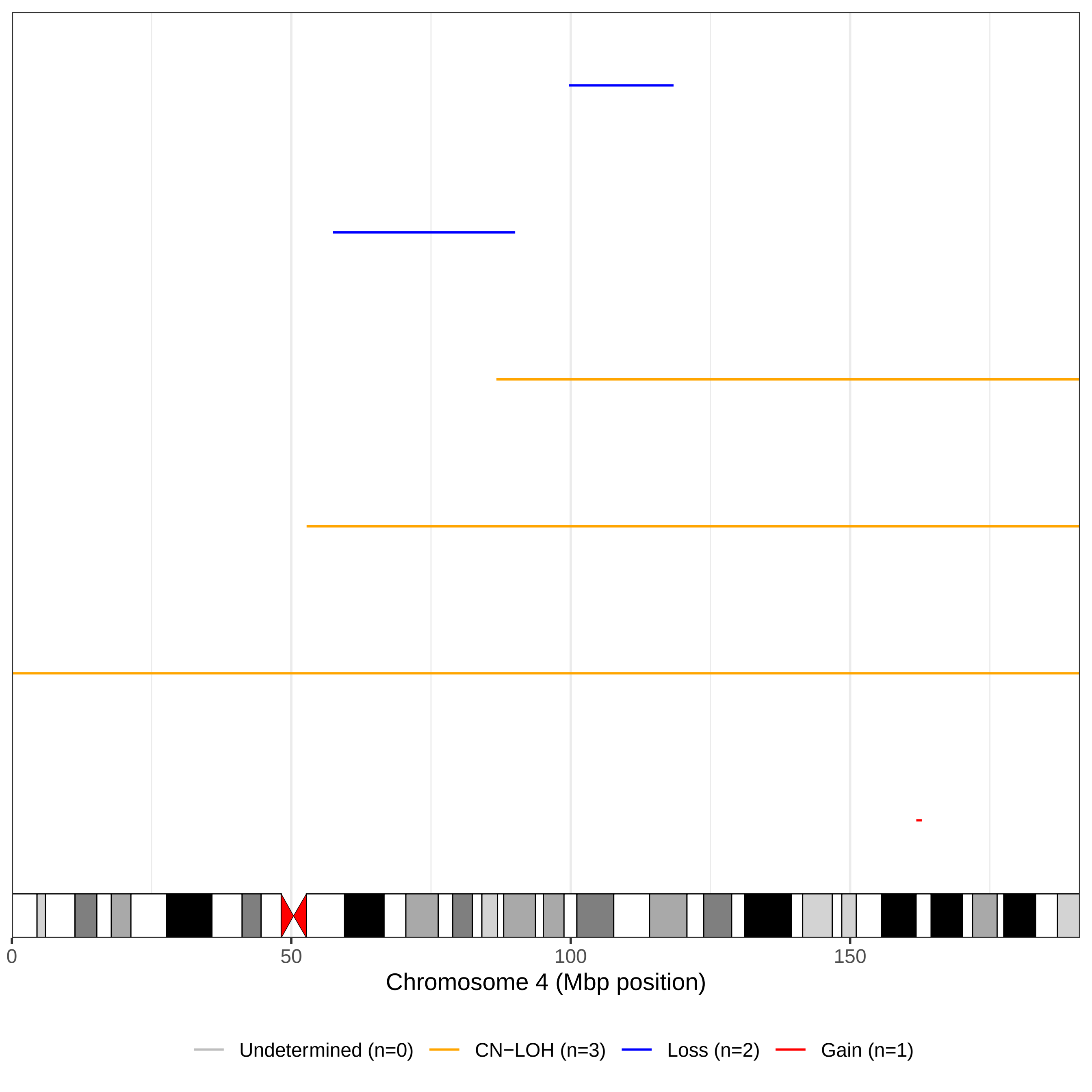

0
50
100
150
Chromosome 4 (Mbp position)
Undetermined (n=0)
CN−LOH (n=3)
Loss (n=2)
Gain (n=1)

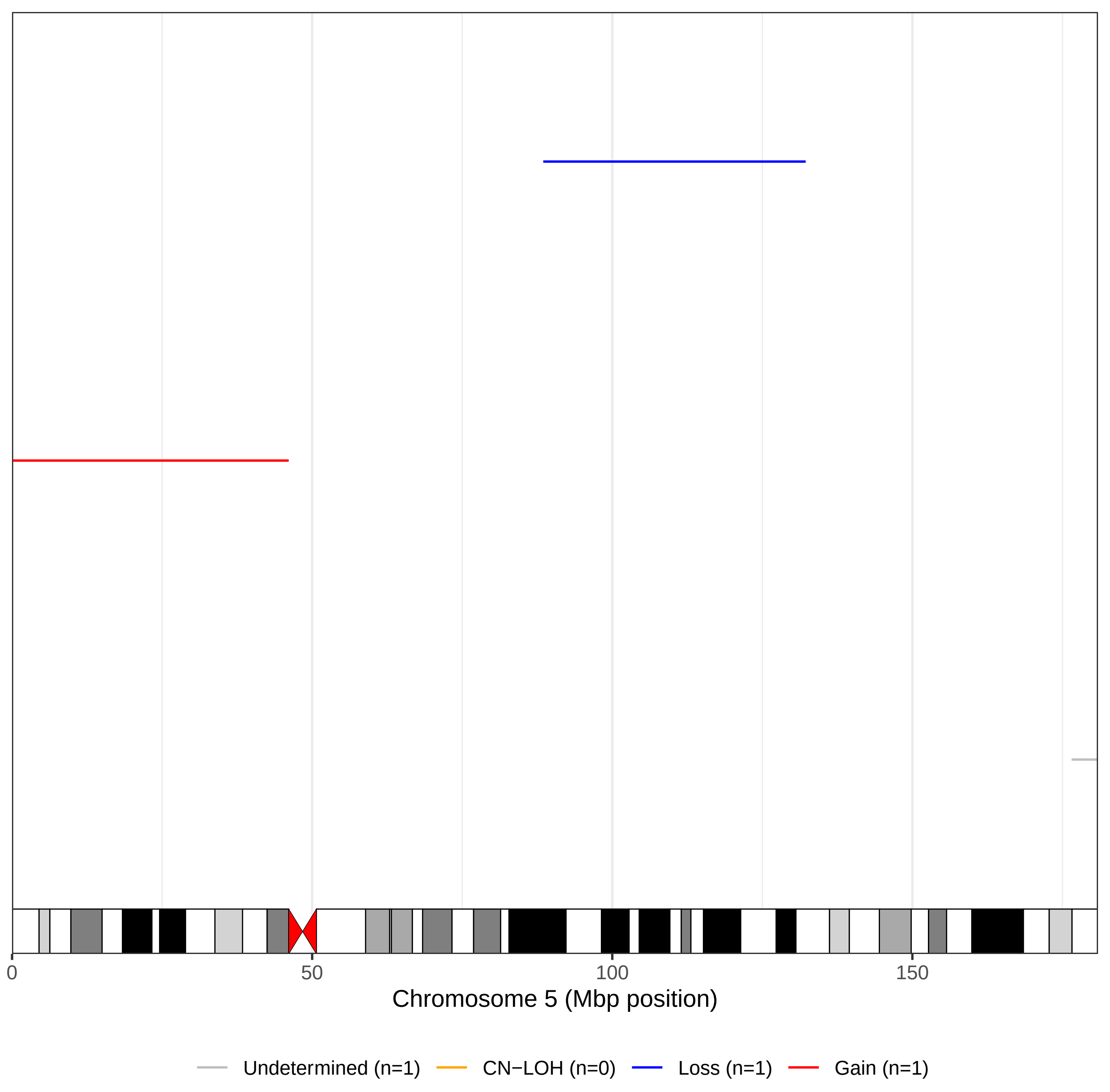
0
50
100
150
Chromosome 5 (Mbp position)
Undetermined (n=1)
CN−LOH (n=0)
Loss (n=1)
Gain (n=1)

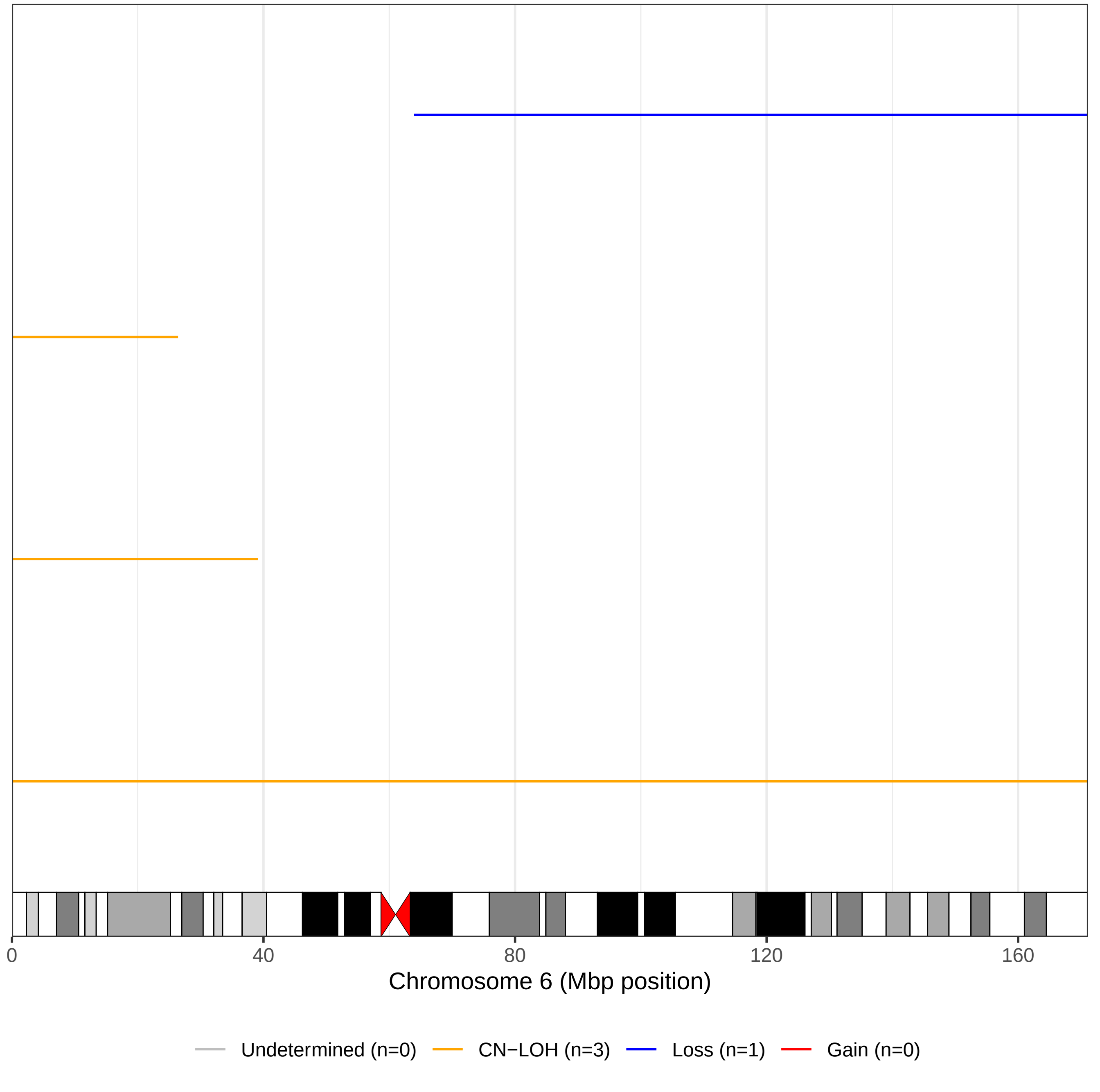

0
40
80
120
160
Chromosome 6 (Mbp position)
Undetermined (n=0)
CN−LOH (n=3)
Loss (n=1)
Gain (n=0)

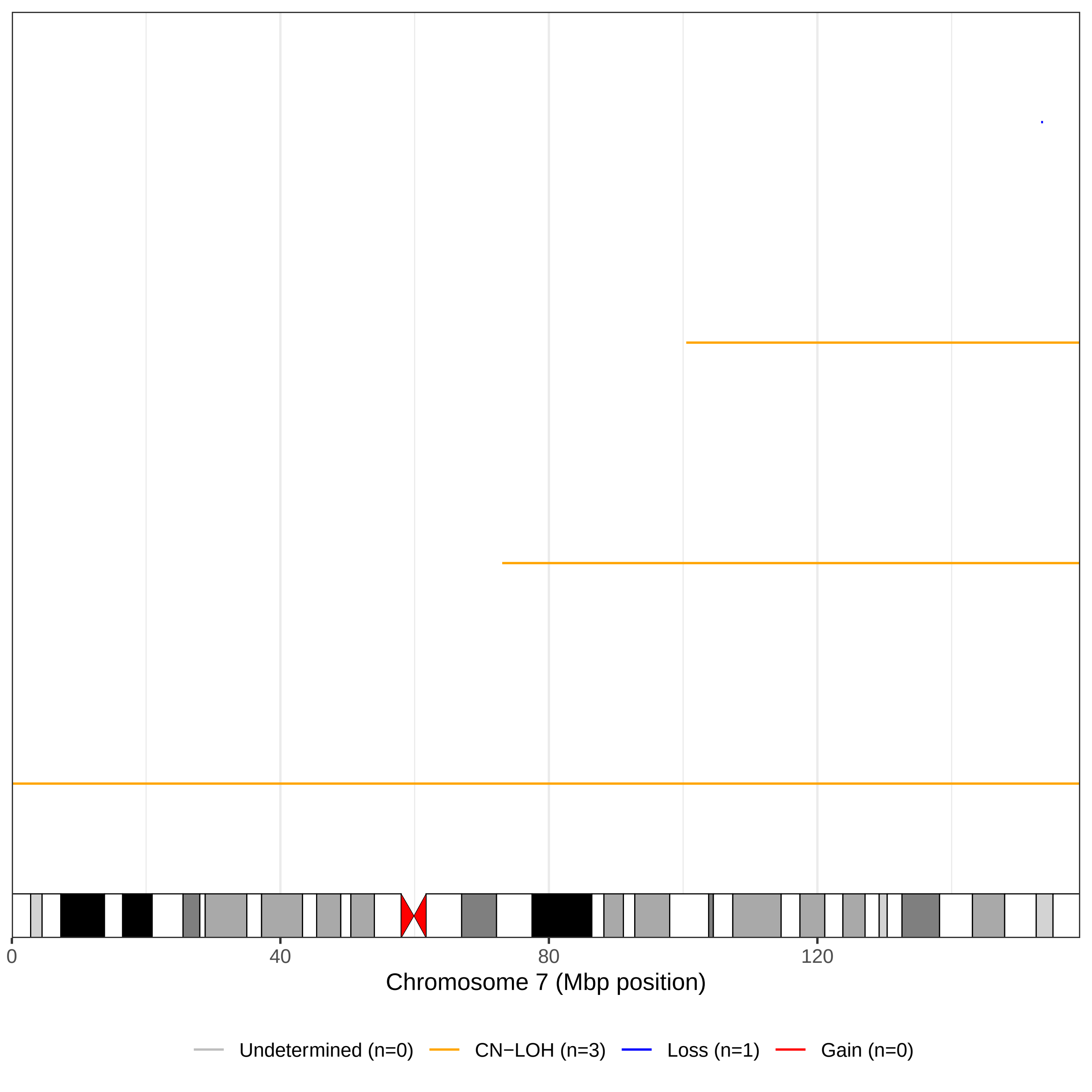
0
40
80
120
Chromosome 7 (Mbp position)
Undetermined (n=0)
CN−LOH (n=3)
Loss (n=1)
Gain (n=0)

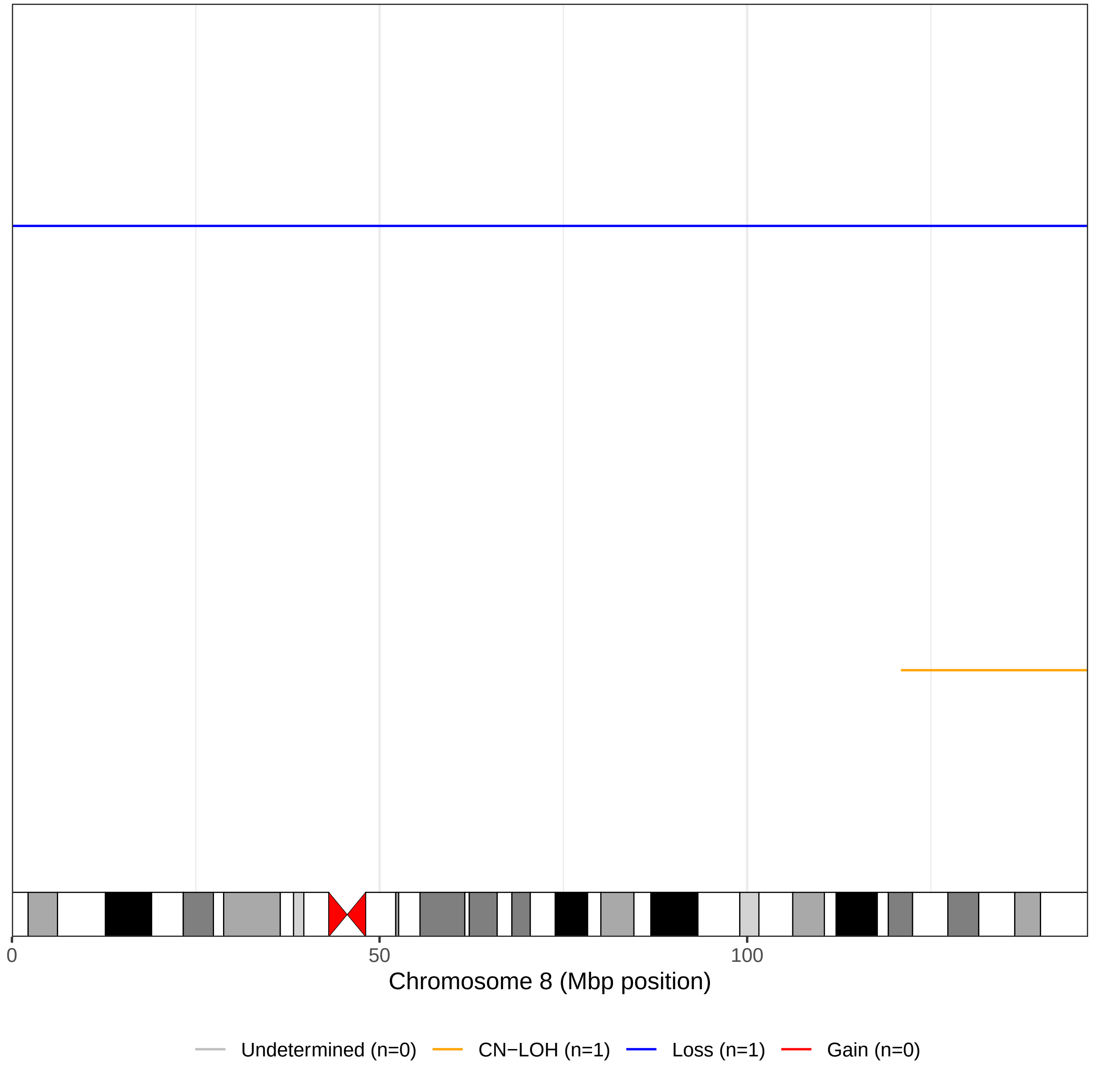
0
50
100
Chromosome 8 (Mbp position)
Undetermined (n=0)
CN−LOH (n=1)
Loss (n=1)
Gain (n=0)

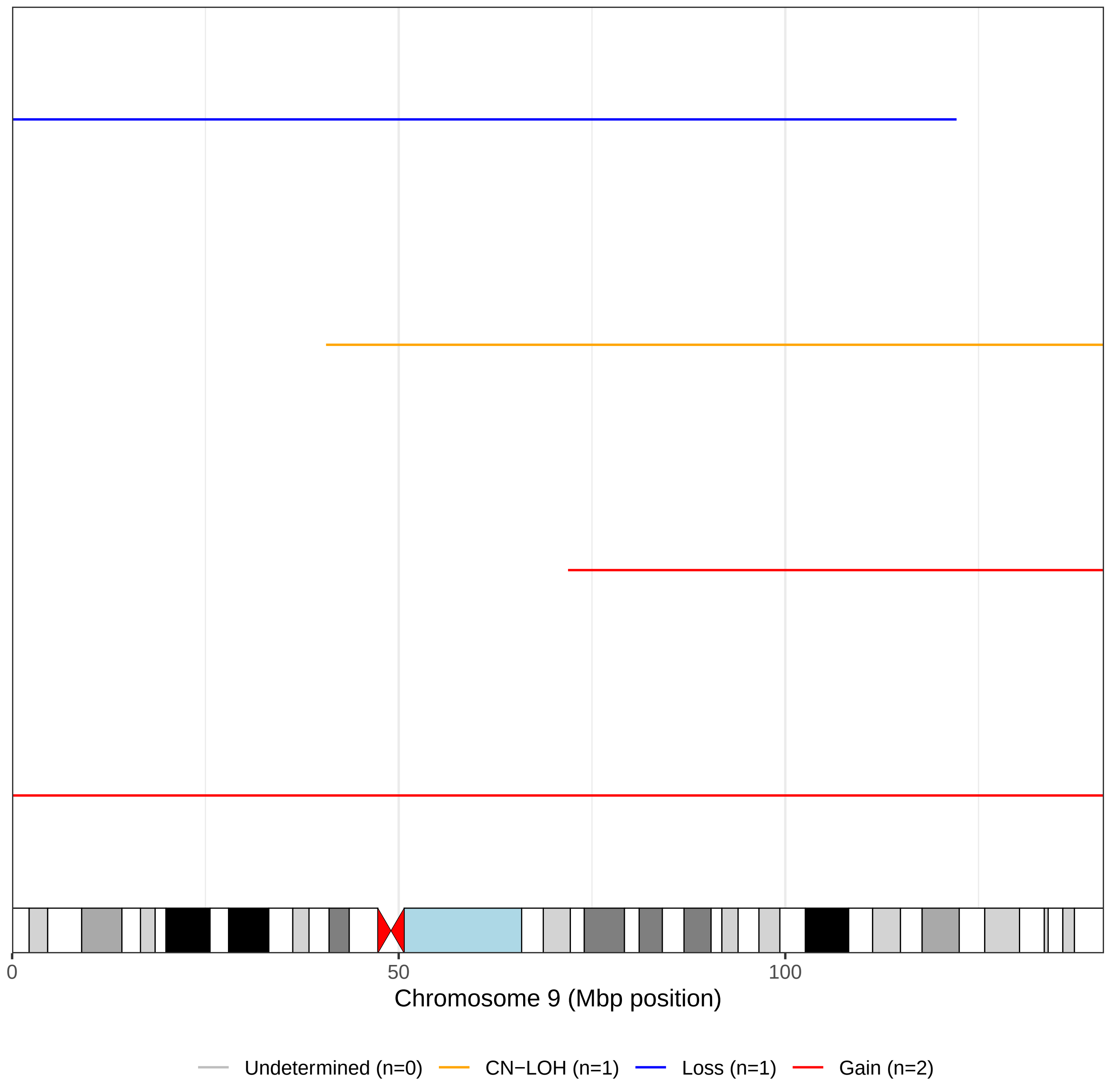

0
50
100
Chromosome 9 (Mbp position)
Undetermined (n=0)
CN−LOH (n=1)
Loss (n=1)
Gain (n=2)

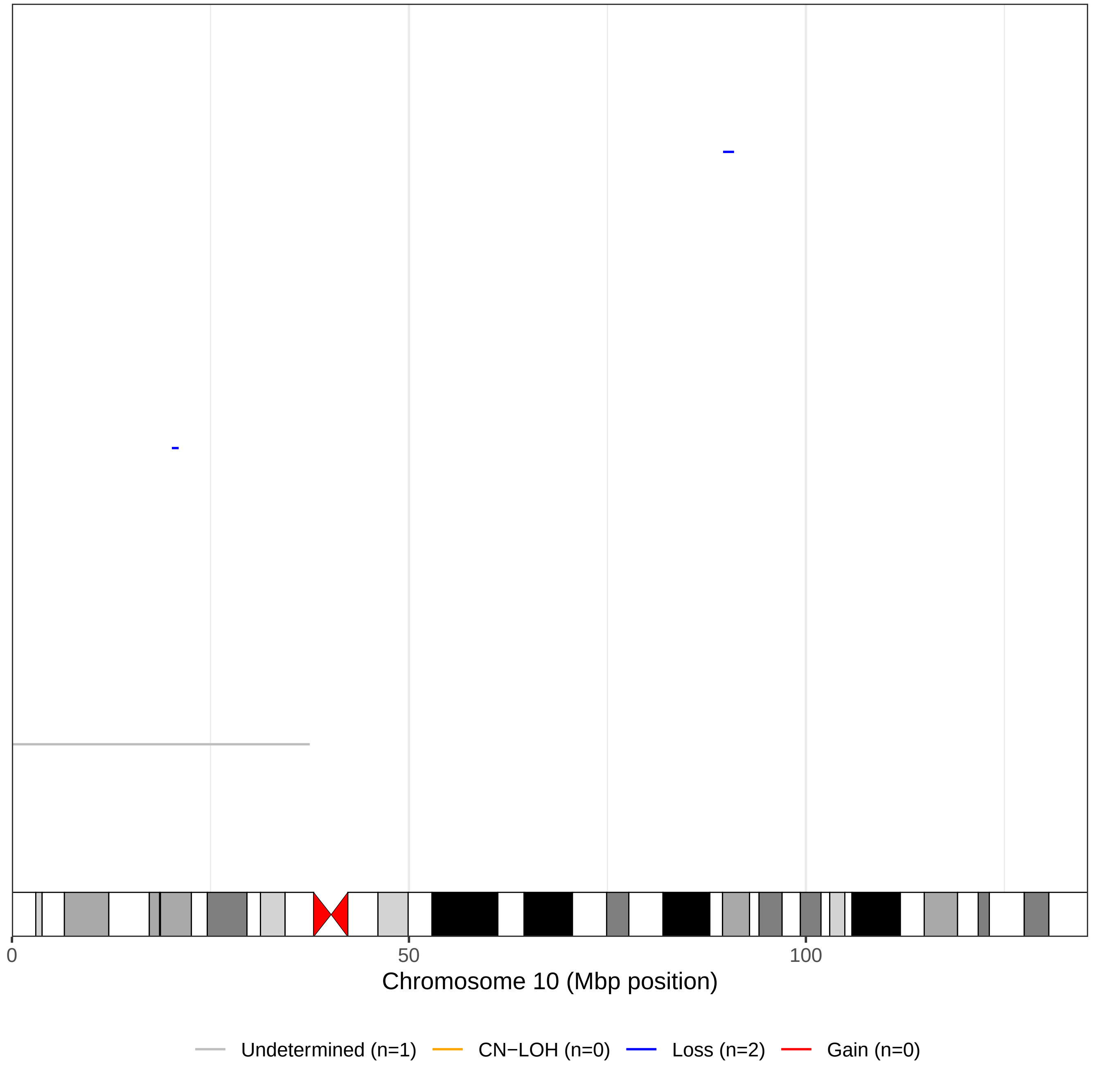
0
50
100
Chromosome 10 (Mbp position)
Undetermined (n=1)
CN−LOH (n=0)
Loss (n=2)
Gain (n=0)

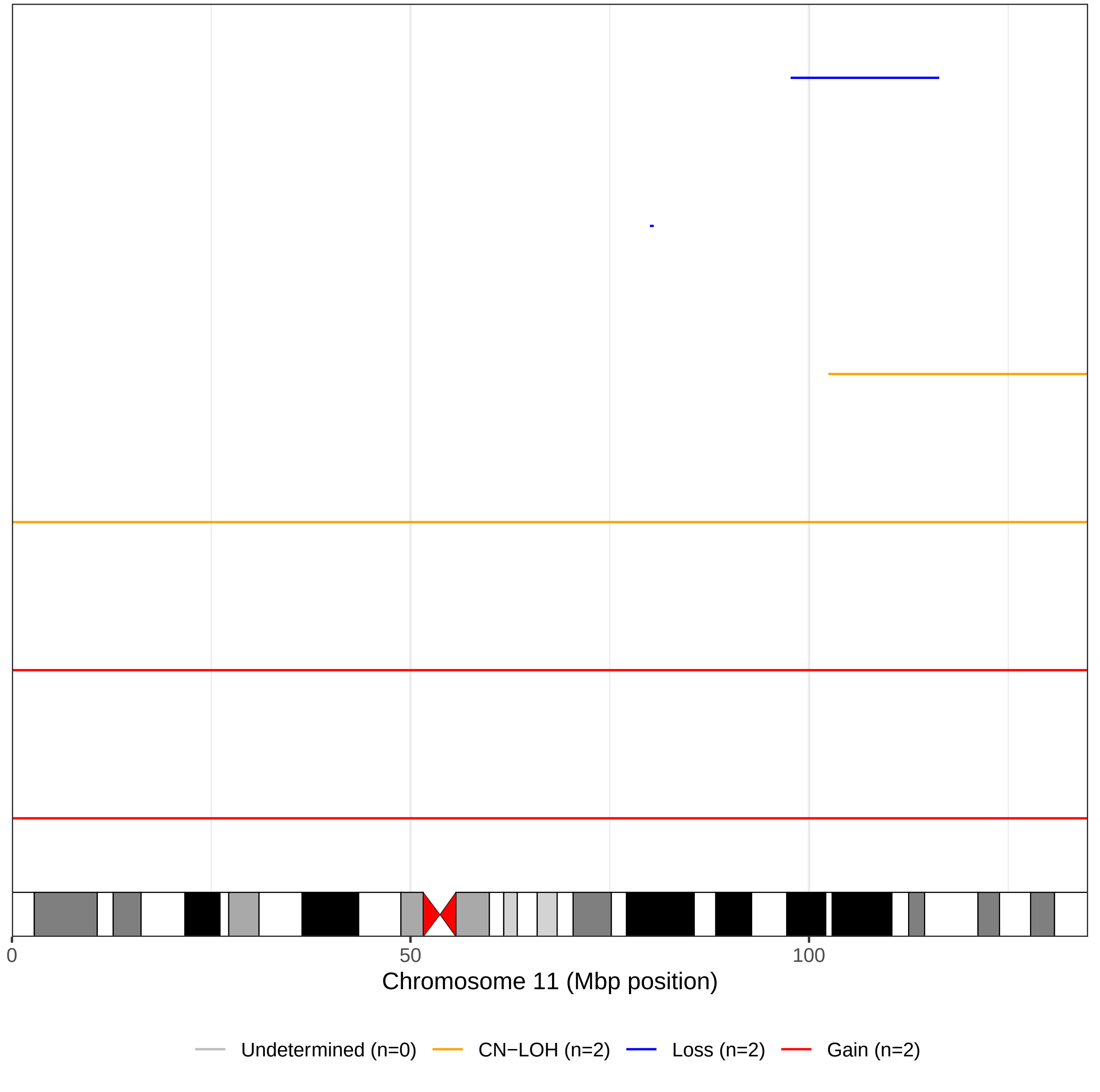

0
50
100
Chromosome 11 (Mbp position)
Undetermined (n=0)
CN−LOH (n=2)
Loss (n=2)
Gain (n=2)

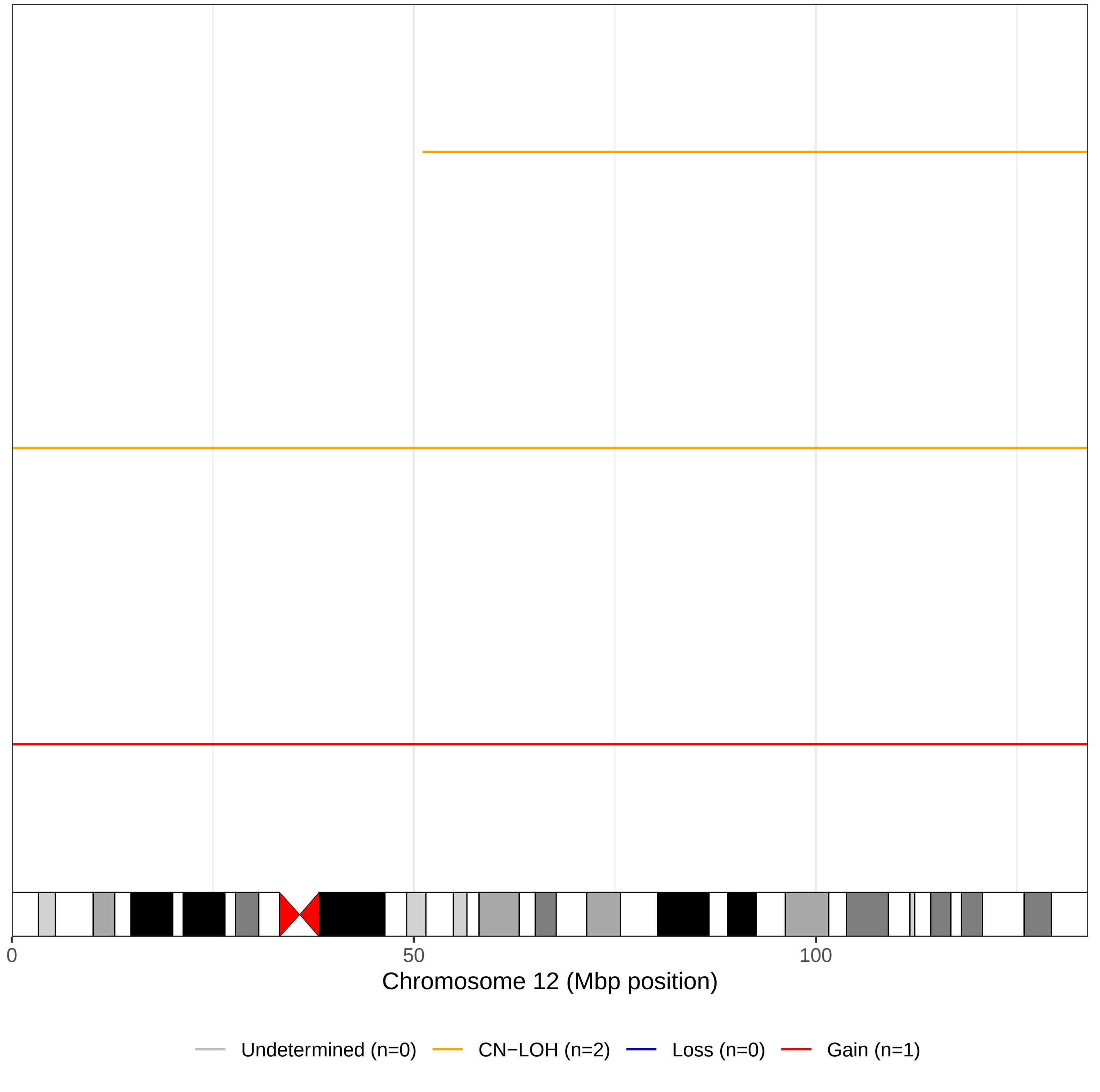
0
50
100
Chromosome 12 (Mbp position)
Undetermined (n=0)
CN−LOH (n=2)
Loss (n=0)
Gain (n=1)

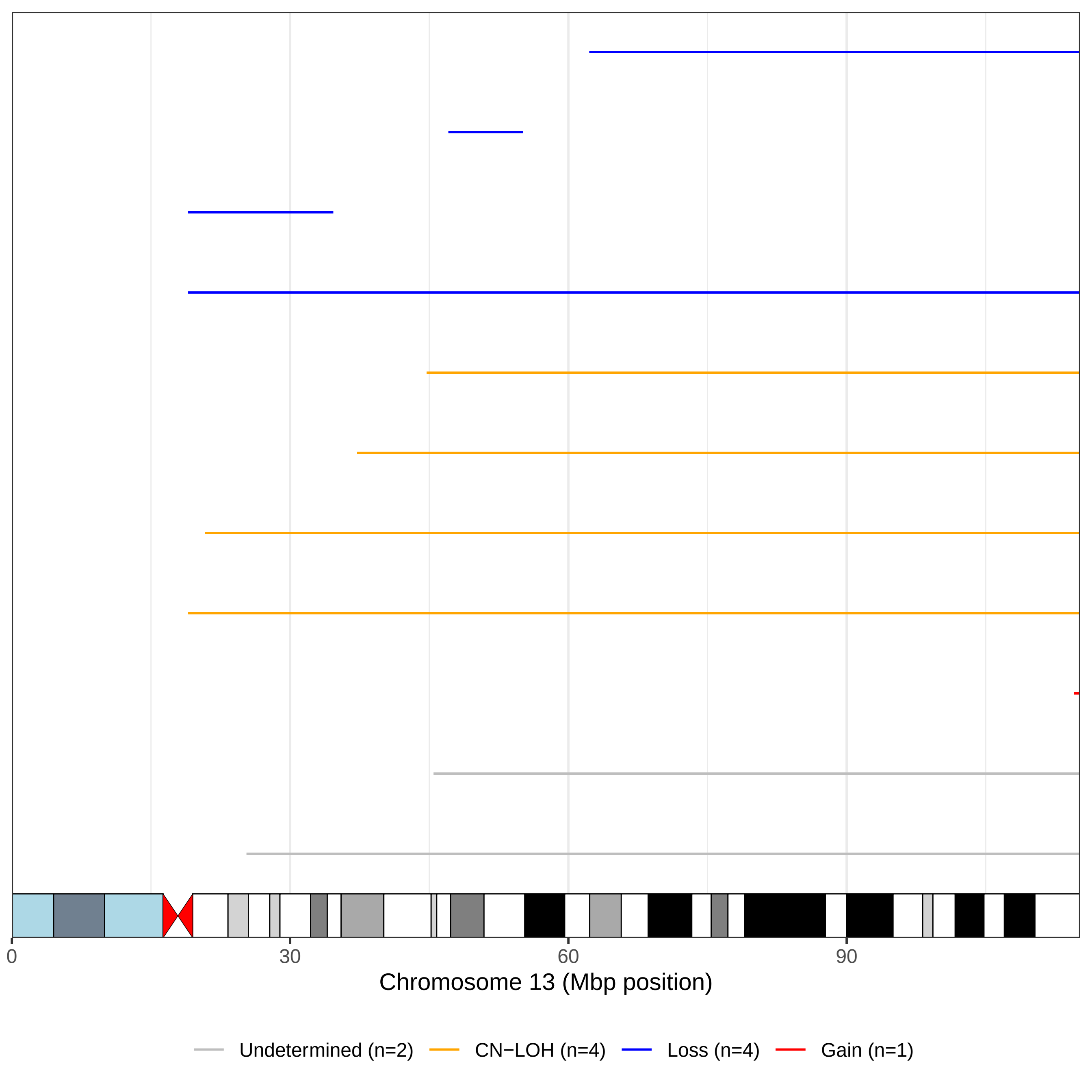
0
30
60
90
Chromosome 13 (Mbp position)
Undetermined (n=2)
CN−LOH (n=4)
Loss (n=4)
Gain (n=1)

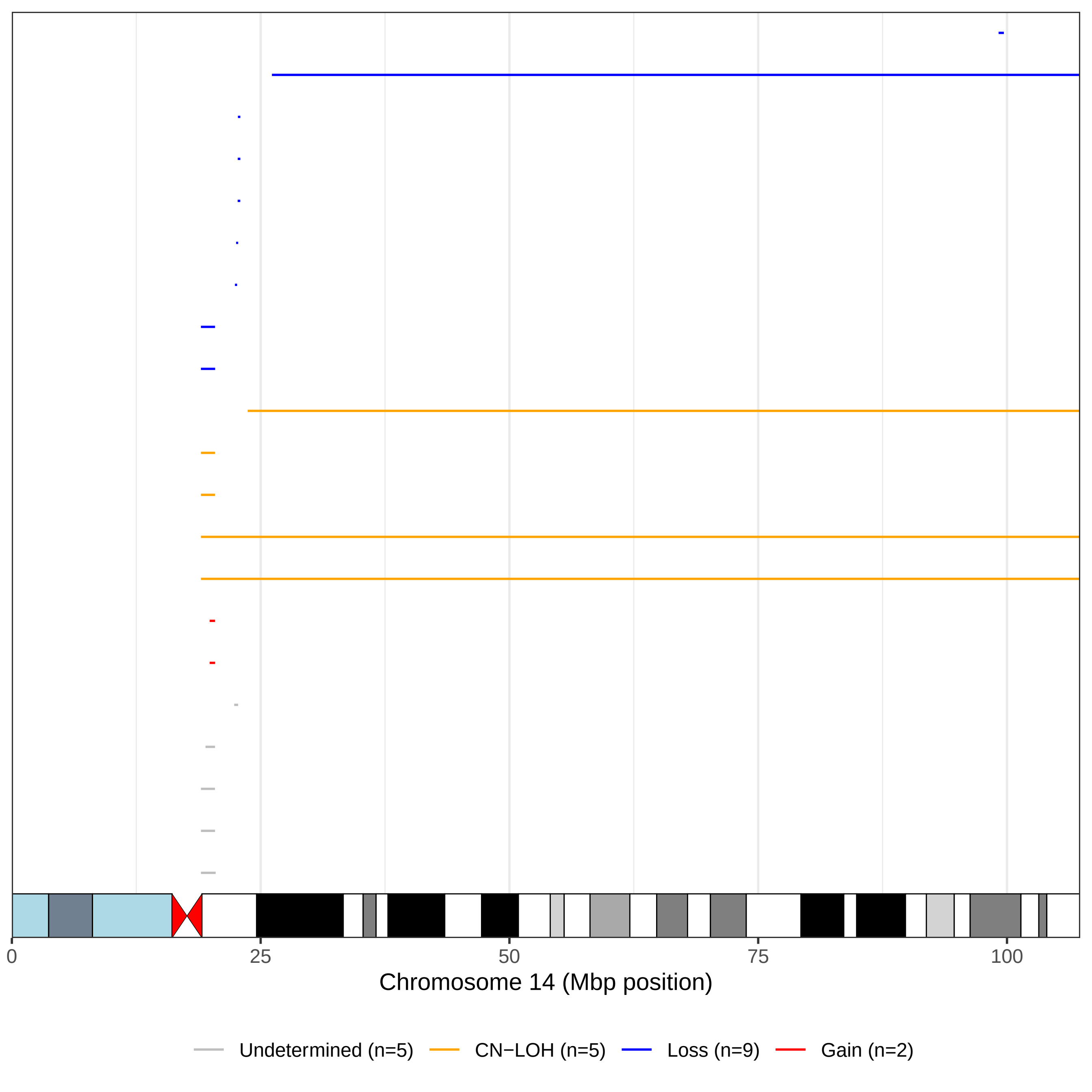

0
25
50
75
100
Chromosome 14 (Mbp position)
Undetermined (n=5)
CN−LOH (n=5)
Loss (n=9)
Gain (n=2)

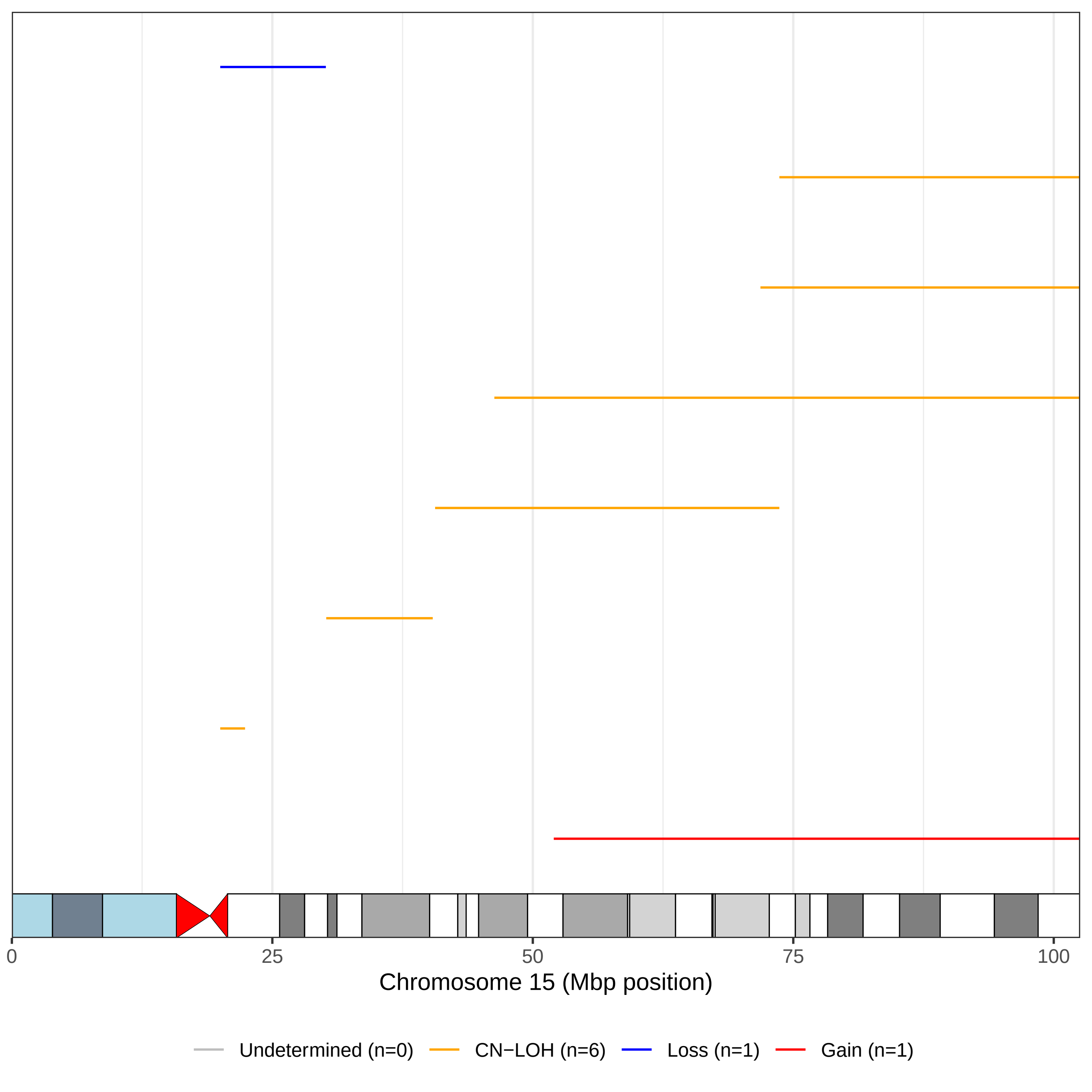
0
25
50
75
100
Chromosome 15 (Mbp position)
Undetermined (n=0)
CN−LOH (n=6)
Loss (n=1)
Gain (n=1)

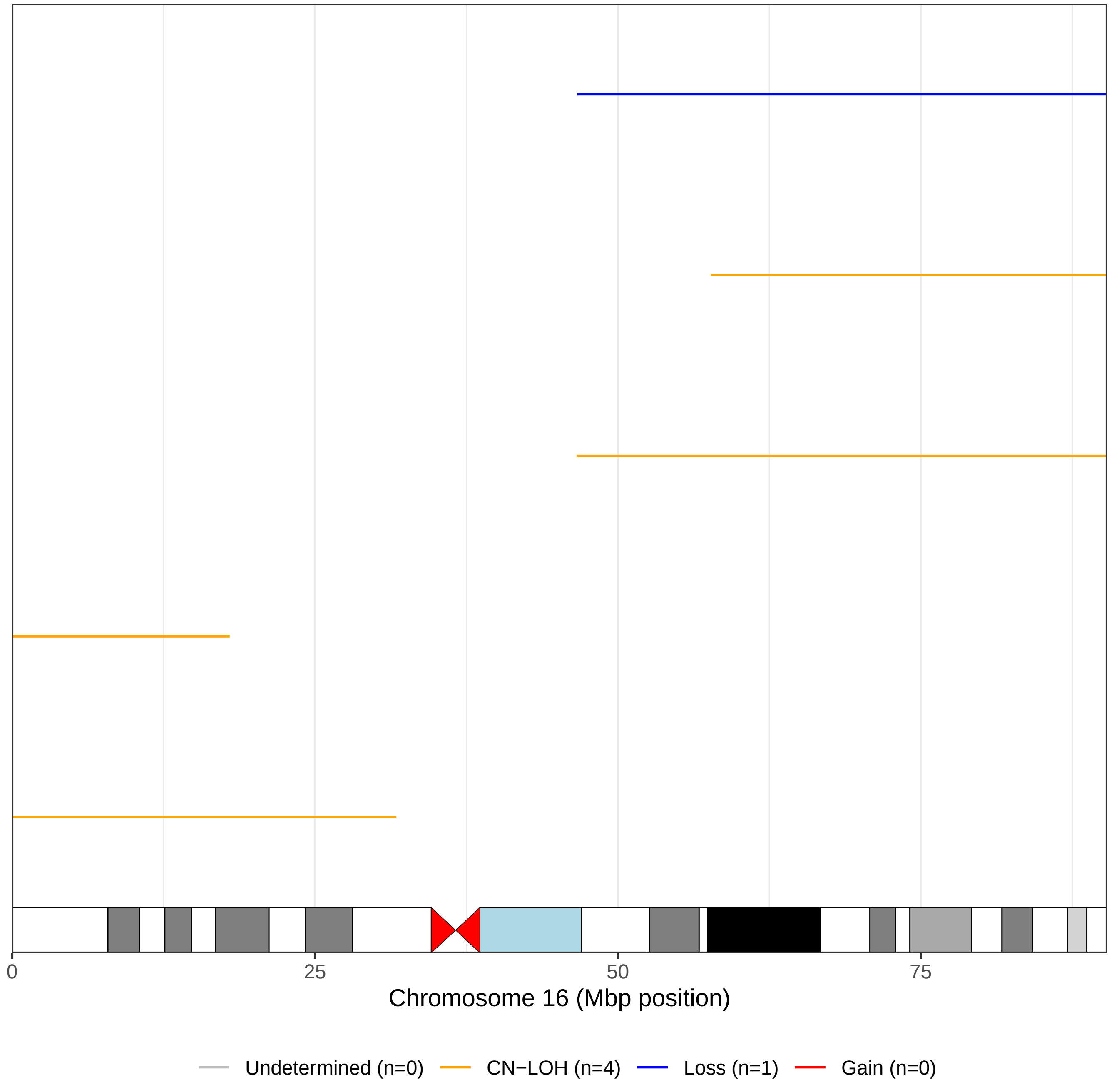
0
25
50
75
Chromosome 16 (Mbp position)
Undetermined (n=0)
CN−LOH (n=4)
Loss (n=1)
Gain (n=0)

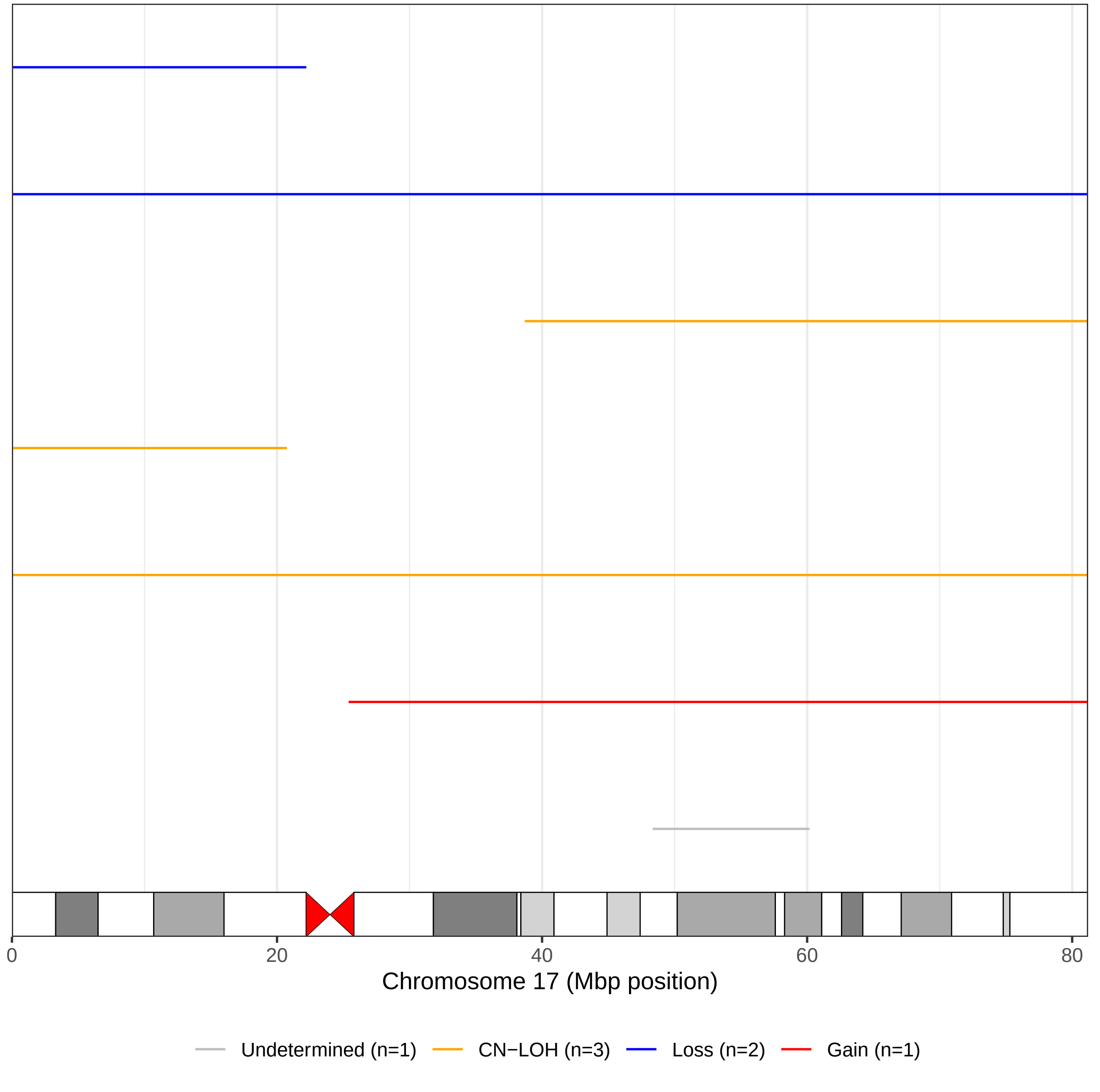
0
20
40
60
80
Chromosome 17 (Mbp position)
Undetermined (n=1)
CN−LOH (n=3)
Loss (n=2)
Gain (n=1)

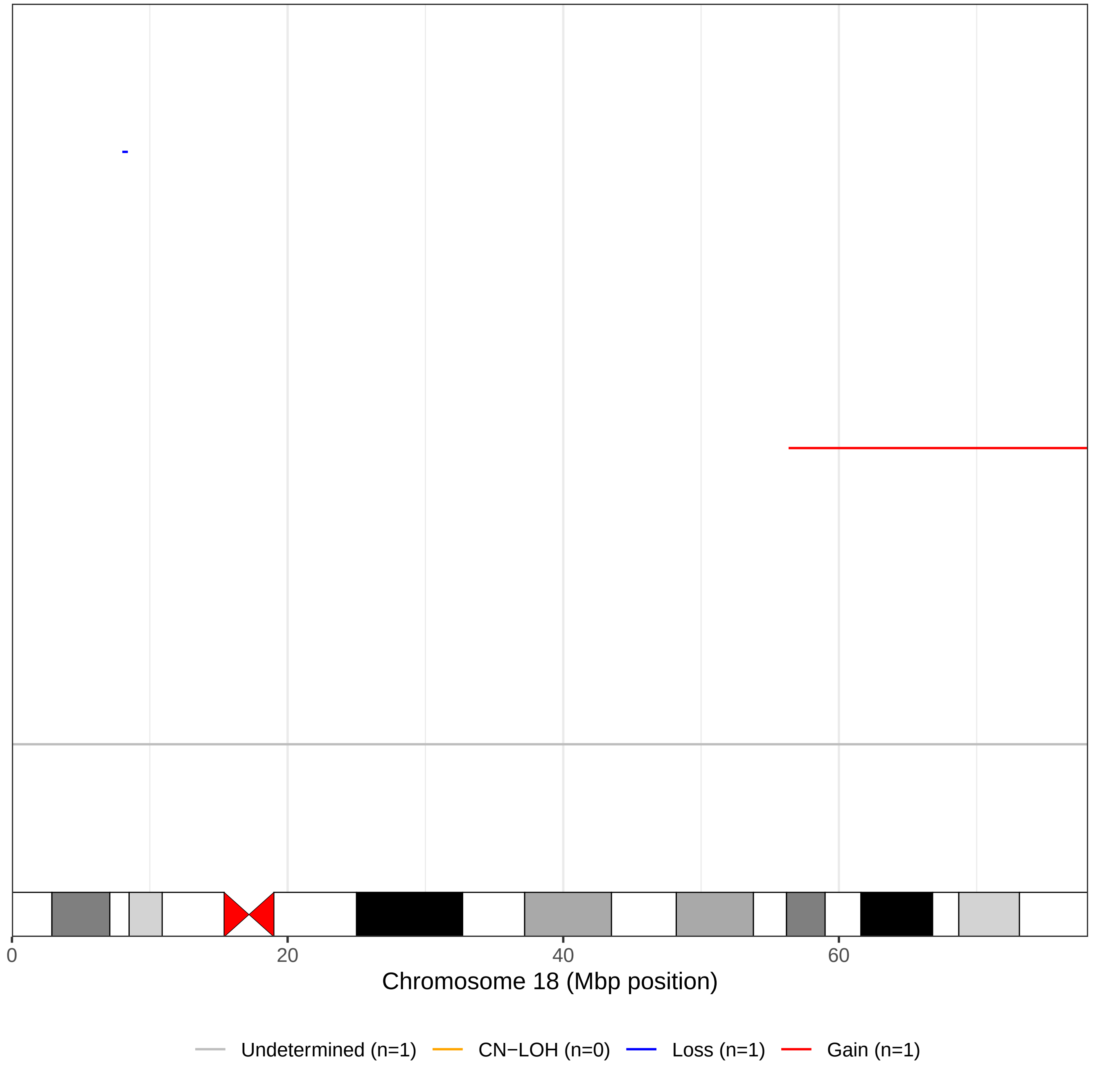
0
20
40
60
Chromosome 18 (Mbp position)
Undetermined (n=1)
CN−LOH (n=0)
Loss (n=1)
Gain (n=1)

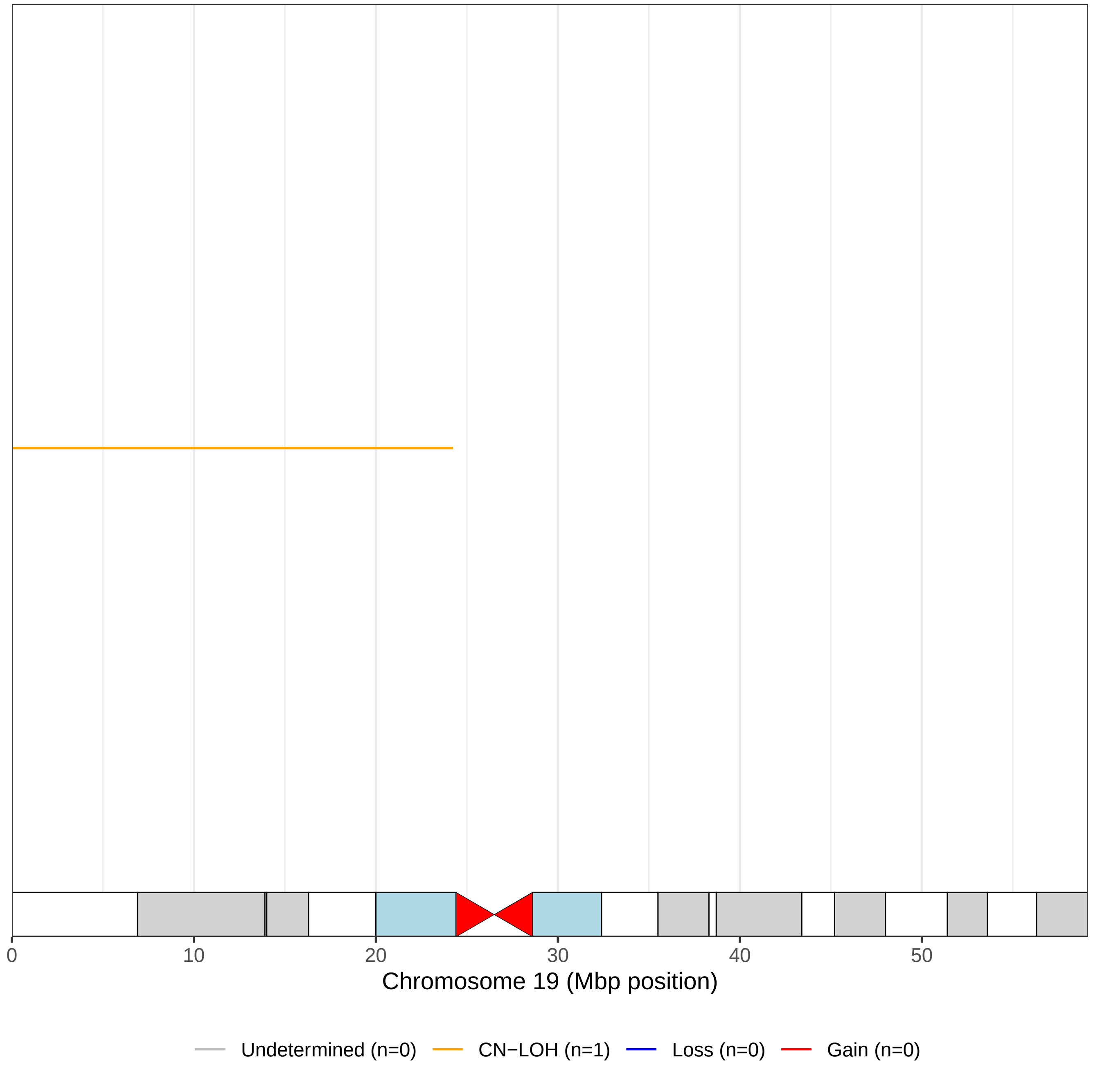
0
10
20
30
40
50
Chromosome 19 (Mbp position)
Undetermined (n=0)
CN−LOH (n=1)
Loss (n=0)
Gain (n=0)

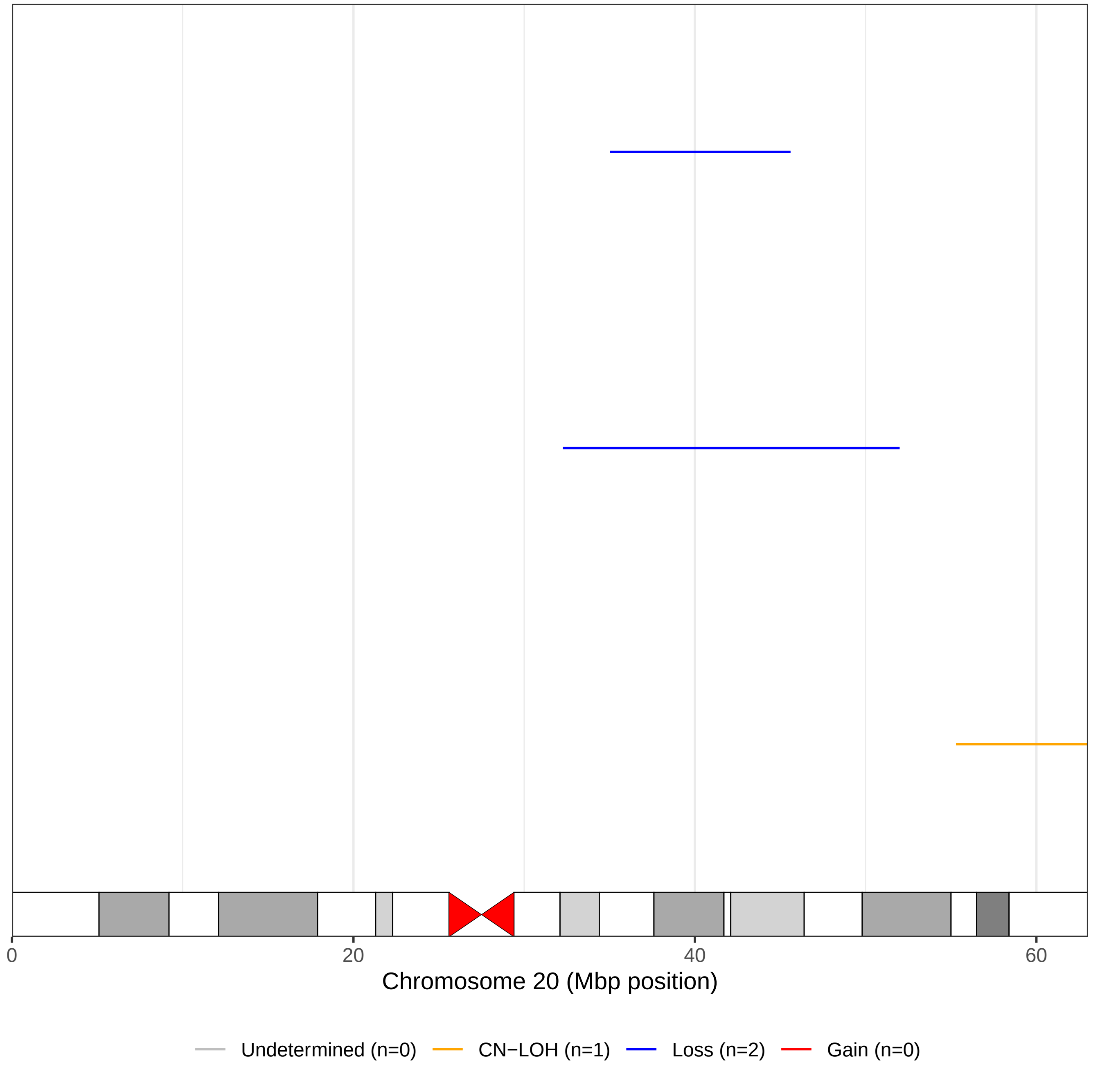
0
20
40
60
Chromosome 20 (Mbp position)
Undetermined (n=0)
CN−LOH (n=1)
Loss (n=2)
Gain (n=0)

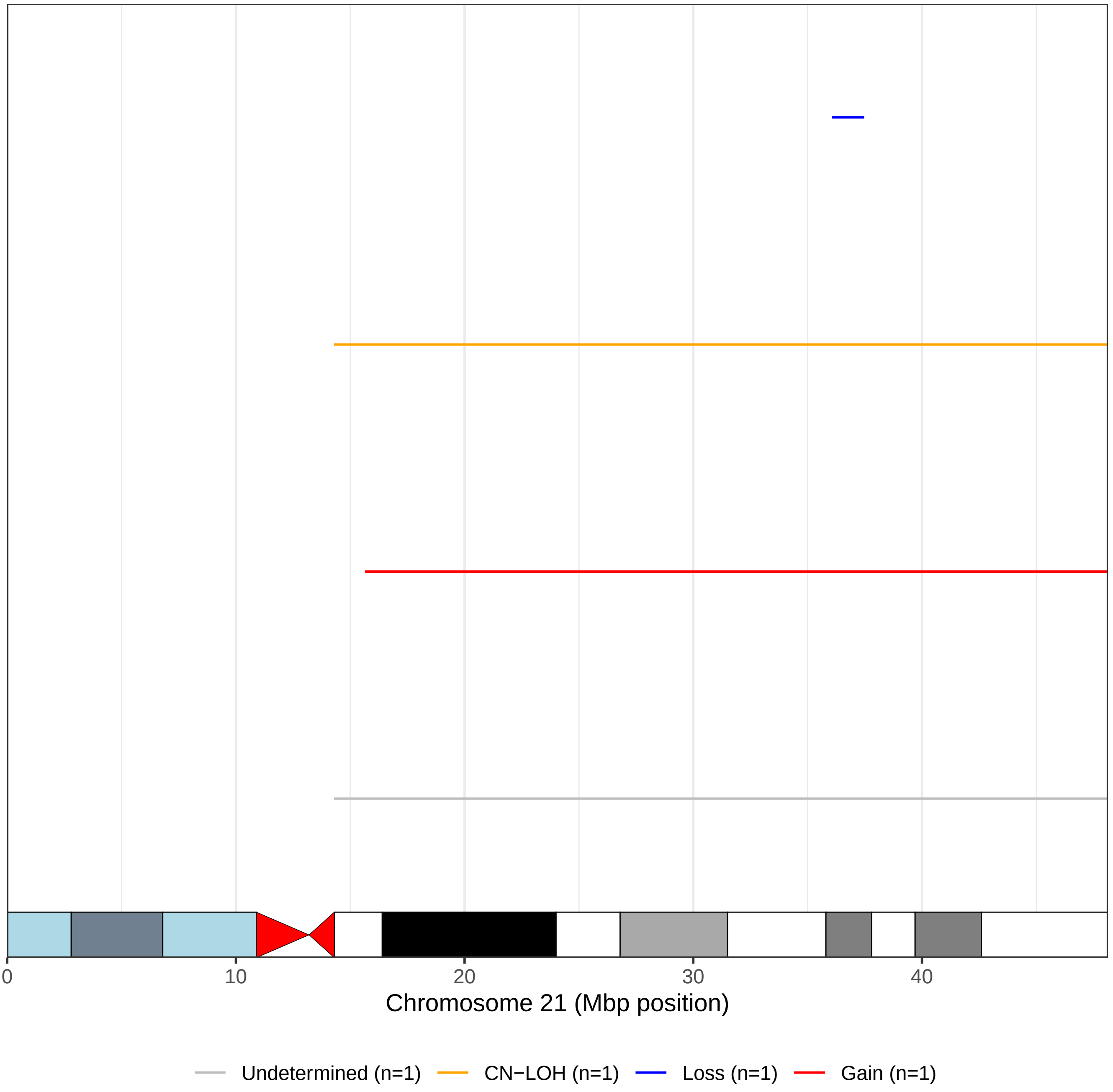
0
10
20
30
40
Chromosome 21 (Mbp position)
Undetermined (n=1)
CN−LOH (n=1)
Loss (n=1)
Gain (n=1)

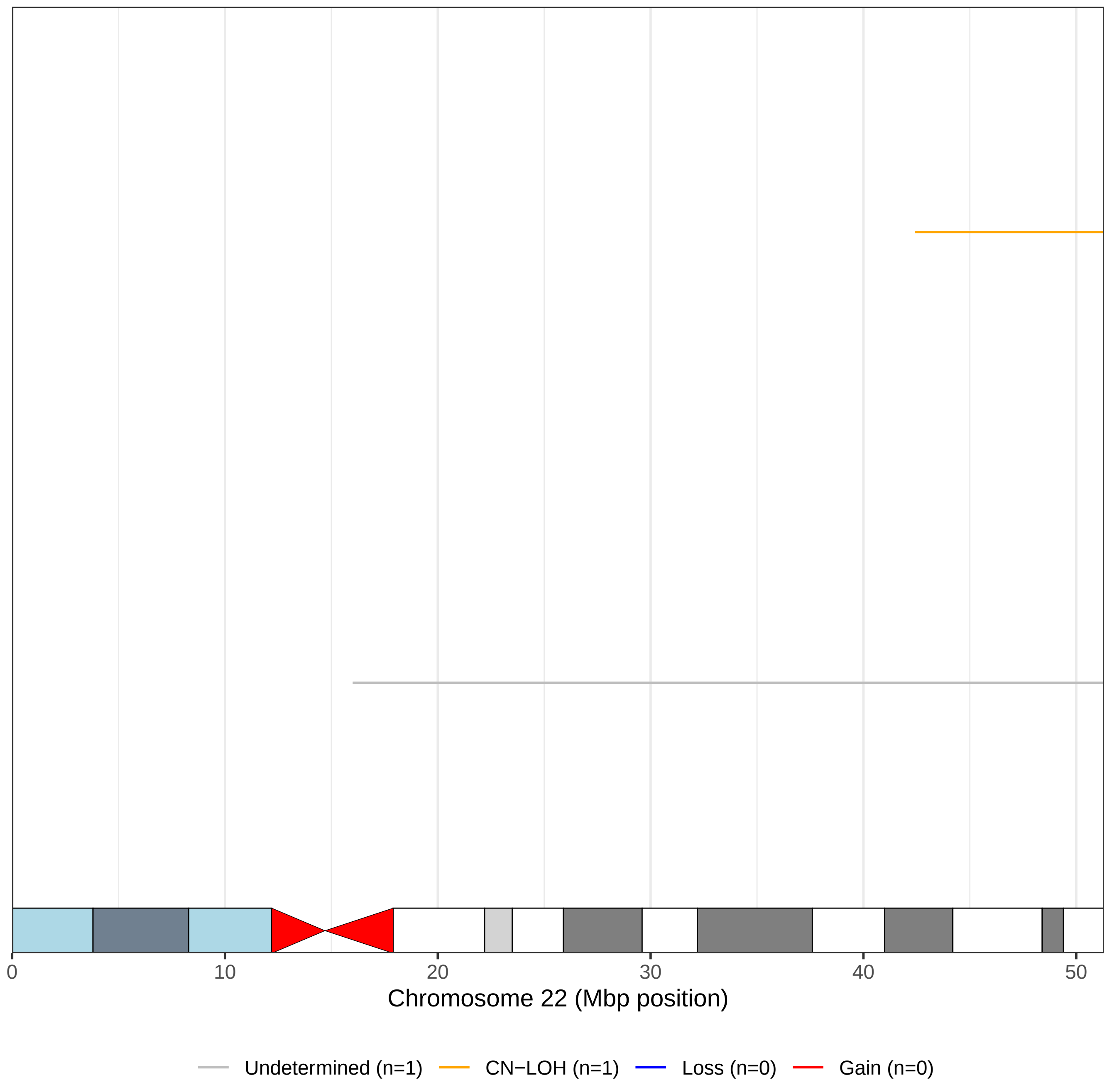
0
10
20
30
40
50
Chromosome 22 (Mbp position)
Undetermined (n=1)
CN−LOH (n=1)
Loss (n=0)
Gain (n=0)

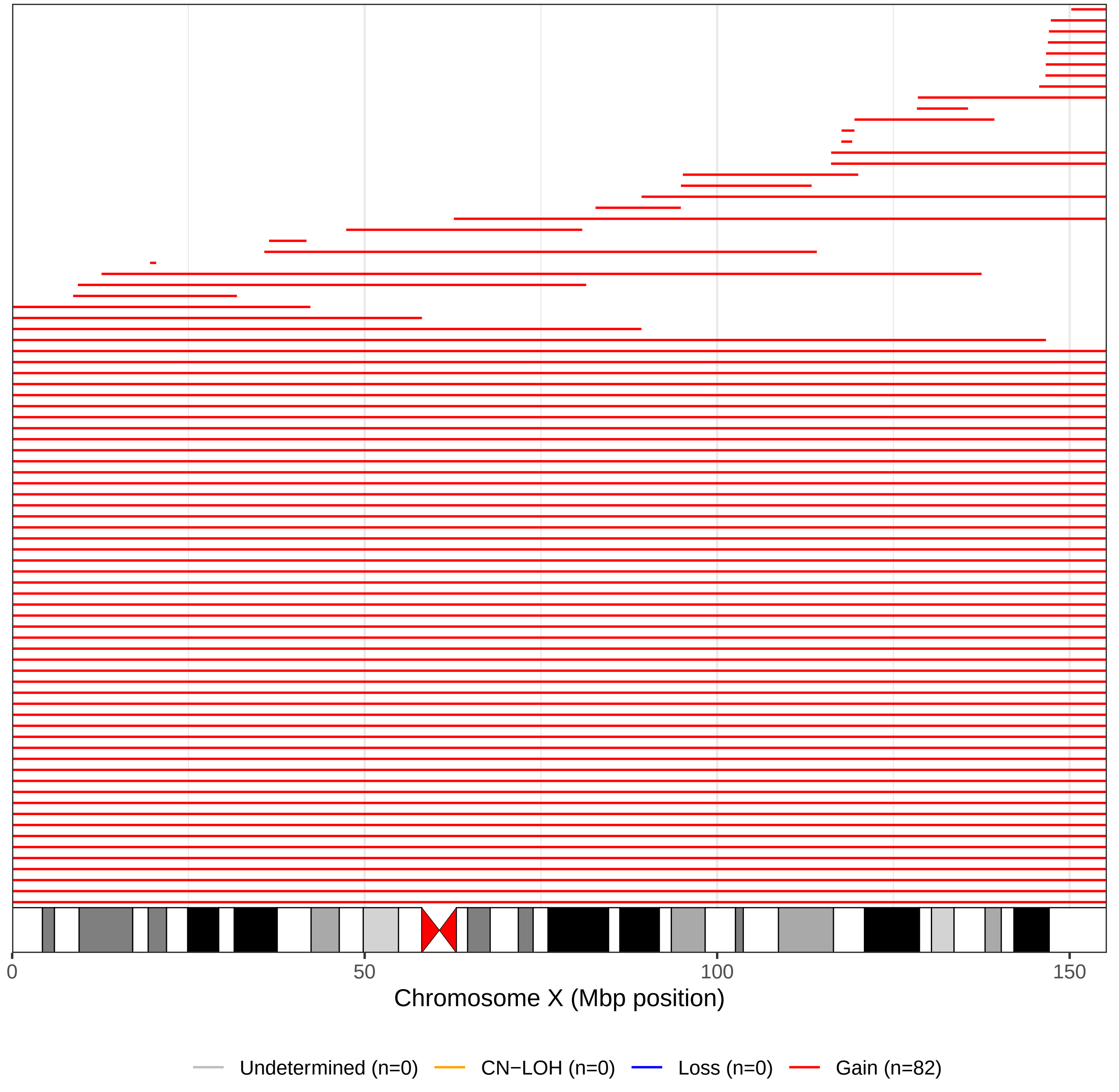
0
50
100
150
Chromosome X (Mbp position)
Undetermined (n=0)
CN−LOH (n=0)
Loss (n=0)
Gain (n=82)
