# Supplementary material for: Mosaic chromosomal alterations in hematopoietic cells and clinical outcomes in patients with multiple myeloma
Source: Leukemia. 2024 Sep 2;38(11):2456–65. doi: 10.1038/s41375-024-02396-3 (PMC11518982; doi:10.1038/s41375-024-02396-3)
Supplement: Supplementary file 5 — Supplemental Note 1 [file 41375_2024_2396_MOESM5_ESM.pdf]

Supplemental Note 1

Infections following ASCT was identified by the following ICD-10 codes:

Infectious disease	ICD-10 codes
**Bacterial pneumonia**	A481, J13-J16, J170, J18, J86
**Sepsis**	A021, A282B, A327, A392-A394, A40-A41, A427, A483, A499A, R572, T802D
**Urinary tract infection**	N109A-N109C, N110-N118B, N118D, N119, N12, N300, N308A-N308C, N309, N390
**Skin infection**	A46, L00-L08, L303, L308F
**Gastroenteritis**	A020, A022-A029, A03-A05, A08-A09
**Other infections**	
Bacterial infection with unspecified location	A490,A498, A499
Osteomyelitis	M86
Herpes	B000-B002C, B005-B009
Endocarditis	I33, I38, I398
Bacterial meningitis	A390, G00-G01, G039, G042
Mycoses	B35-B49
Hepatitis	B15-B19, Z225
Imported & parasitic infections	A00-A01, A06-A07, A90-A96, B50-B64
Viral meningitis	A87, B003,B004, B004A,B020-DB021 G020
Influenza and viral lower respiratory tract infections	J09-J101C, J12, J171
HIV/AIDS	B20-B24, F024, Z21
Tuberculosis	A15-A19, N330, N740-N741
Parasitic worm diseases	B65-B83, N308J

Information on variables and data from the Danish National Multiple Myeloma Registry (DaMyDa) can be found in following openly accessible article:

Gimsing P, Holmström MO, Klausen TW, Andersen NF, Gregersen H, Pedersen RS, et al. The Danish National Multiple myeloma registry. Clin Epidemiol. 2016;8:583–7.

https://www.rkkp-dokumentation.dk/Public/PDF/GetFile.aspx?Db=74&FileID=14285
